# Transcriptional and metabolomic analysis of *Ascophyllum nodosum* mediated freezing tolerance in *Arabidopsis thaliana*

**DOI:** 10.1186/1471-2164-13-643

**Published:** 2012-11-21

**Authors:** Prasanth Nair, Saveetha Kandasamy, Junzeng Zhang, Xiuhong Ji, Chris Kirby, Bernhard Benkel, Mark D Hodges, Alan T Critchley, David Hiltz, Balakrishnan Prithiviraj

**Affiliations:** 1 Department of Environmental Sciences, Dalhousie Agricultural Campus, Dalhousie University, Truro, NS, B2N 5E3, Canada; 2 Institute for Nutrisciences and Health, National Research Council of Canada, Charlottetown, PEI, C1A 4P3, Canada; 3 Crops and Livestock Research Centre, Agriculture and Agri-Food Canada, 550 University Avenue, Charlottetown, PE, C1A 4N6, Canada; 4 Atlantic Food and Horticulture Research Centre, Agriculture and Agri-Food Canada, Kentville, NS, B4N 1J5, Canada; 5 Acadian Seaplants Limited, 30 Brown Ave., Dartmouth, NS, B3B 1X8, Canada

**Keywords:** *Arabidopsis thaliana*, *Ascophyllum nodosum*, Freezing tolerance, Chemical priming, Soluble sugars, Metabolite profiling, Microarray analysis

## Abstract

**Background:**

We have previously shown that lipophilic components (LPC) of the brown seaweed *Ascophyllum nodosum* (ANE) improved freezing tolerance in *Arabidopsis thaliana*. However, the mechanism(s) of this induced freezing stress tolerance is largely unknown. Here, we investigated LPC induced changes in the transcriptome and metabolome of *A. thaliana* undergoing freezing stress.

**Results:**

Gene expression studies revealed that the accumulation of proline was mediated by an increase in the expression of the proline synthesis genes *P5CS1* and *P5CS2* and a marginal reduction in the expression of the proline dehydrogenase (*ProDH*) gene. Moreover, LPC application significantly increased the concentration of total soluble sugars in the cytosol in response to freezing stress. Arabidopsis *sfr4* mutant plants, defective in the accumulation of free sugars, treated with LPC, exhibited freezing sensitivity similar to that of untreated controls. The ^1^H NMR metabolite profile of LPC-treated Arabidopsis plants exposed to freezing stress revealed a spectrum dominated by chemical shifts (δ) representing soluble sugars, sugar alcohols, organic acids and lipophilic components like fatty acids, as compared to control plants. Additionally, 2D NMR spectra suggested an increase in the degree of unsaturation of fatty acids in LPC treated plants under freezing stress. These results were supported by global transcriptome analysis. Transcriptome analysis revealed that LPC treatment altered the expression of 1113 genes (5%) in comparison with untreated plants. A total of 463 genes (2%) were up regulated while 650 genes (3%) were down regulated.

**Conclusion:**

Taken together, the results of the experiments presented in this paper provide evidence to support LPC mediated freezing tolerance enhancement through a combination of the priming of plants for the increased accumulation of osmoprotectants and alteration of cellular fatty acid composition.

## Background

Environmental factors such as low temperature, drought, and salinity are the major abiotic stress factors that adversely affect the growth and development of plants limiting crop productivity [1]. Freezing stress affects plant growth directly by the inhibition of metabolic reactions and indirectly through osmotic, oxidative and other secondary stresses.

A number of chemicals have been tested for inducing freezing tolerance. Whereas exogenous application of cryoprotectants such as sorbitol and polyethylene glycol were found to be either phyto-toxic or only marginally effective in imparting frost tolerance [2], chemicals such as choline chloride and ethanolamine have been shown to be effective in improving frost tolerance, in wheat and tomato seedlings, respectively [3,4]. More recently, some acrylic compounds were found to protect crops from freezing damage by forming an inert layer on the plant surface [2]. However, the environmental safety of these chemicals and their impact on various ecosystems is unclear. Therefore, the identification of naturally occurring plant-based products to impart freezing stress tolerance to crop plants would be ideal.

Extracts of *Ascophyllum nodosum*, have been shown to stimulate shoot growth and branching [5], increase root growth and lateral root development [6], improve nutrient uptake [7], enhance resistance to diseases [8], and mitigate the effects of environmental stresses such as drought, salinity and frost [9,10]. Burchett et al. [11] reported that application of a commercial formulation of *A. nodosum* extract enhanced winter hardiness and increased frost resistance in winter barley. Another study in grapes using an extract of the Tasmanian Giant Bull kelp *Durvillea potatorum* (Labill.)] also improved plant freezing tolerance [12]*.*

The role of proline in low temperature tolerance in plants has been reported [13-15] and a number of plant species, that are inherently tolerant to freezing temperatures (such as barley, rye, winter wheat, grape, potato and Arabidopsis), accumulate high levels of proline when exposed to low temperatures [16,17]. Proline plays multiple roles in frost tolerance; such as a mediator of osmotic adjustment [18], stabilizer of proteins and membranes [19], inducer of osmotic stress-related genes [20], scavenger of reactive oxygen species (ROS) [21], source of reduction equivalents during stress recovery [22], and readily available source of nitrogen and carbon during post-recovery growth [23].

In an earlier study, we demonstrated that *A. nodosum* extracts (ANE) and its lipophilic component (LPC) significantly enhanced freezing tolerance in *Arabidopsis thaliana*[24]. Electrolyte leakage measurements revealed that the LT_50_ value of LPC treated plants was lowered by 3°C while cell viability staining demonstrated a 30-40% reduction in area of damaged tissue in extract treated plants as compared to water controls. Additionally, ANE treatment caused a two-fold increase in the transcription of the cold response genes, *cor15a, rd29a* and transcription factor *cbf3.*

Several expression profile studies in Arabidopsis have established that large scale changes occur in gene expression during cold acclimation and subsequent freezing tolerance [25-27]. Freezing temperatures activate a number of cold-responsive genes which encode a diverse array of proteins such as dehydrins, lipid transfer proteins, molecular chaperones, anti-freeze proteins, enzymes involved in respiration and metabolism of carbohydrates, phenylpropanoids and antioxidants, late-embryogenesis-abundant proteins and others, each with a presumed function in tolerance to the dehydration caused by freezing [28-32].

Microarrays provide an advantage of allowing parallel quantification of the gene expression at the whole genome level. Here, we report changes in the metabolome and global transcript profile in plants, after treatment with the LPC and subsequent exposure to freezing temperatures and post-freezing recovery.

## Results

### Proline estimation

**Figure 1:**
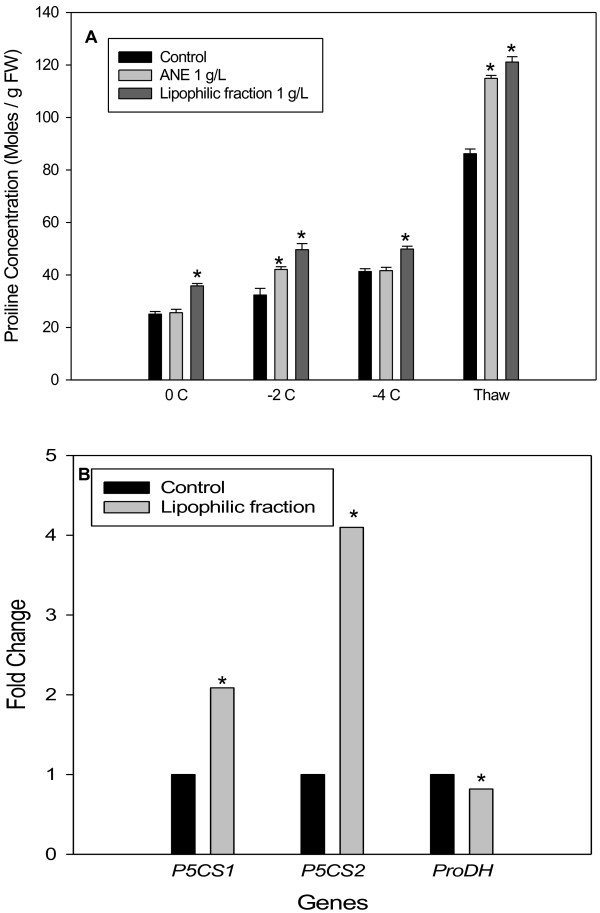
**Proline accumulation and expression of proline metabolism genes in wild-type (WT) *Arabidopsis*: (A) effect of ANE and LPC on the accumulation of proline in the leaves of wild type Arabidopsis (Col-0) in response to freezing stress (Data are the means ± SE) and (B) Real time-PCR analysis of expression of genes encoding Δ**^**1**^**-pyrroline-5-carboxylate synthetase (*P5CS*) and prolinedehydogenase (*ProDH*).** Bar with * is significantly different (P ≤ 0.05) from that of the control treatment.

Plants treated with ANE or LPC accumulated higher amounts of proline during freezing stress, as compared to water controls (Figure 1A). Freezing stress was initiated by spraying plants with ice-cold water in an incubator set at 0°C and progressively cooled at the rate of 1°C per 24h until desired temperature was reached. The proline content of control plants after exposure to a freezing stress of 0°C for 24 h was 25.06 micro moles g^-1^ fresh weight versus 35.82 micro moles g^-1^ fresh weight in LPC treated plants. At this temperature, plants treated with ANE did not show a significant change in proline content, as compared to control plants. However, when plants were exposed to −2°C for 24 h, ANE- and LPC-treated plants accumulated higher amounts of proline (42.11 and 49.62 micro moles g^-1^ fresh weight, respectively) as compared to the untreated plants (32.32 moles g^-1^ fresh weight). A further lowering of temperature to −4°C did not significantly affect proline accumulation in any of the treatments. During thawing, Arabidopsis plants accumulated large amounts of proline amounting to 86.20, 114.89 and 121.10 micro moles g^-1^ fresh weight in control, ANE- and LPC-treated plants, respectively. Overall, LPC treated plants accumulated significantly higher amounts of proline at all temperatures (Figure 1A).

### Transcriptional analysis of proline biosynthesis and degradation genes

Analysis of transcript abundance for genes involved in proline biosynthesis (*P5CS1* and *P5CS2*) and degradation (*ProDH*) in −2°C treatments revealed that ANE and LPC treatment affected the expression of these genes (Figure 1B). There was a two-fold increase in *P5CS1* and a four-fold increase in *P5CS2* transcripts as compared to plants that did not receive LPC treatment. A marginal decrease in the expression of *ProDH* was also observed (Figure 1B).

### Proline mutant studies

**Figure 2:**
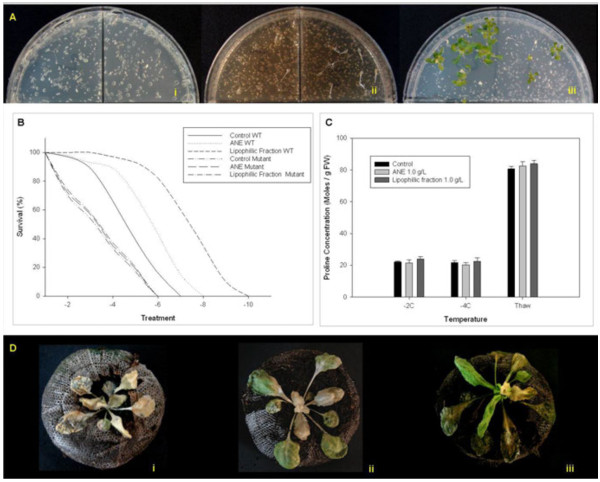
***Ascophyllum nodosum *extracts did not rescue the freezing sensitive phenotype of *p5cs1 *mutants: (A) Petri Plate Freezing Tolerance Assay with wild-type Arabidopsis (left partition of Petri plate) and *p5cs1 *mutants (right partition of Petri plate) treated with (i) Control (ii) *Ascophyllum nodosum *extract (ANE) and (iii) lipophilic component of ANE (LPC), (B) survival rate at different freezing temperatures of wild type Arabidopsis and *p5cs1 *mutant plants treated with ANE or LPC in Petri Plates, (C) proline accumulation of *p5cs1 *mutants treated with ANE or LPC in response to freezing in peat pellets, and (D) Phenotypic responses of *p5cs1 *mutant plants treated with (i) water control (ii) ANE (1.0 g L**^**-1**^**) and (iii) LPC (1.0 g L**^**-1**^**) to a temperature of −2°C for 24 h in Peat pellet freezing assay.** Bars are the means ± SE.

The role of proline in ANE-mediated freezing tolerance in Arabidopsis, was confirmed by using *p5cs-1* mutants, deficient in proline accumulation during stress. Observations of *p5cs-1* mutants revealed that application of ANE and LPC did not alter the sensitive phenotype of *p5cs-1* mutants (Figure 2). When the temperature was lowered to −7.5°C, the wild-type, water control plants showed 100% mortality, while the wild-type plants treated with LPC showed considerably less damage and were able to recover from stress-induced damage (Figure 2).

### Soluble sugar estimation and sugar mutant studies

**Figure 3:**
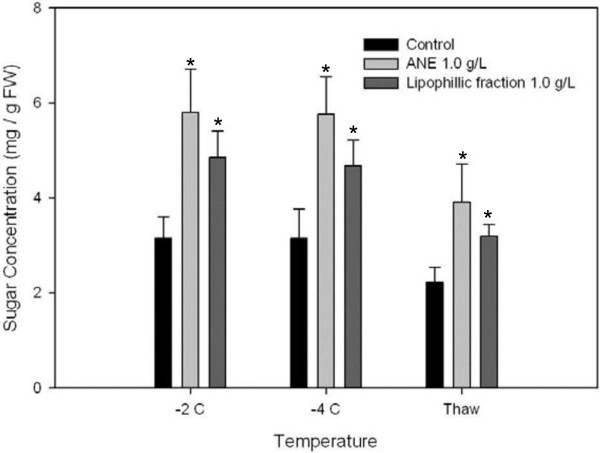
**Accumulation of soluble sugars in wild-type Arabidopsis plants treated with ANE or LPC under different freezing and thawing regimes.** Data are the means ± SE, Bar with * is significantly different (P ≤ 0.05) from that of the control treatment.

Wild-type plants treated with ANE or LPC accumulated higher amounts of total soluble sugars in response to freezing stress, as compared to untreated controls (Figure 3). At -2°C, ANE-treated plants showed an almost two-fold increase in soluble sugars (5.79 mg g^-1^ fresh weight) while plants treated with LPC showed a 1.5-fold increase (4.89 mg g^-1^ fresh weight) as compared to untreated controls (3.15 mg g^-1^ fresh weight). Levels of soluble sugars remained unchanged in the treated and control plants at −4°C. Soluble sugar concentrations were significantly higher in treated plants (3.91 and 3.20 mg g^-1^ fresh weight of ANE- and LPC-treated plants, respectively), as compared to untreated controls (2.22 mg g^-1^ fresh weight) (Figure 3).

### Confirmation of the role of soluble sugars in ANE-mediated freezing tolerance using Arabidopsis mutant *sfr4*

**Figure 4:**
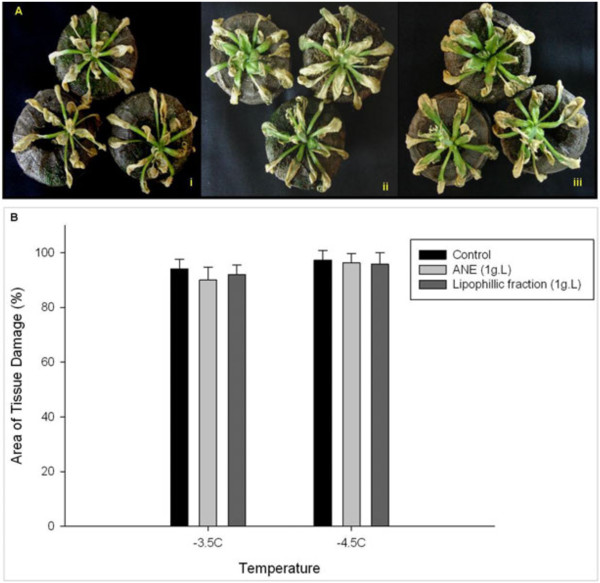
**Sugar accumulation is required for ANE-mediated freezing tolerance in *Arabidopsis*: (A) sensitive phenotype of *sfr4 *mutant plants treated with (i) water control (ii) ANE (1.0 g L**^**-1**^**) and (iii) LPC (1.0 g L**^**-1**^**) to a temperature of −2.5°C for 24 h in Peat Pellet Freezing Tolerance Assay, and (B) extent of freezing-induced tissue damage in controls and treated *sfr4 *mutant plants as revealed by trypan blue staining; comparison of the area of tissue damage in the trypan blue stained leaves using the image processing and analysis software Image-j®.** Data are means ± SE.

The extent of tissue damage in *sfr4* mutants revealed that ANE or LPC failed to impart freezing tolerance to plants which are defective in sugar accumulation (Figure 4). Mutant plants treated with ANE or LPC showed similar levels of damage to untreated controls when exposed to freezing temperatures (Figure 4A). At −3.5°C, 92% of the leaf area of control plants was damaged, compared to 90% and 87% in ANE- and LPC-treated plants, respectively. The percentage area of tissue damage was 96% and 95%, respectively, for ANE and LPC treated plants at a temperature of −4.5°C, while it was 97% for control plants (Figure 4B). These results confirm that sugar accumulation is essential in the development of ANE-mediated freezing tolerance in Arabidopsis.

### ^1^H NMR metabolite profiles of ANE extracts

**Figure 5:**
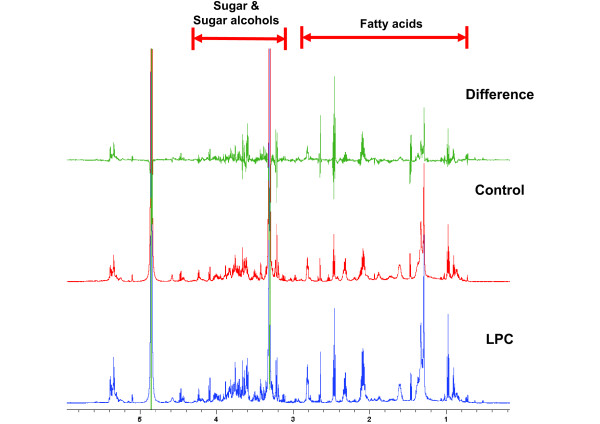
^**1**^**H-NMR metabolite profile spectrum of wild-type Arabidopsis plants treated with LPC during freezing temperature of −2°C for 24 h.**

**Figure 6:**
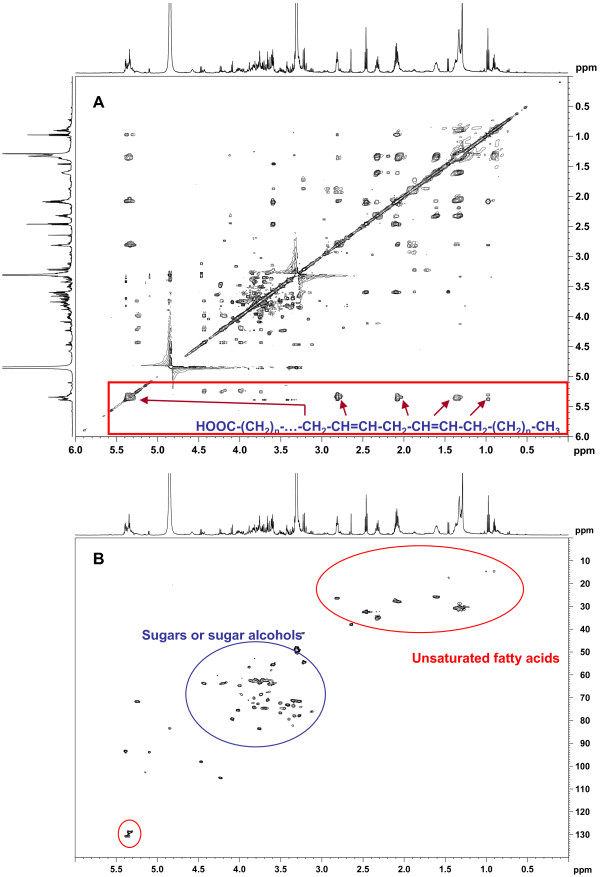
2D NMR spectra of metabolites extracted from LPC-treated plants, upon exposure to freezing stress of −2.5°C for 24 h: (A) 2D NMR TOCSY spectrum showing characteristic proton correlations in unsaturated fatty acids and (B) 2D NMR HSQC spectrum showing characteristic proton and carbon signals of unsaturated fatty acids and sugars or sugar alcohols.

Application of LPC elicited significant changes in the metabolome of Arabidopsis in response to freezing stress. At ambient temperature, the application of ANE or LPC had minimal effect on the metabolic profile of Arabidopsis. However, when exposed to −2°C for 24 h, significant increases in peaks with ^1^H NMR chemical shifts (δ) at 0.8 to 1.6, 2.1 to 2.8, 3.2 to 3.9, and 5.2 to 5.5 ppm were observed in the plants treated with LPC, as compared to control (Figure 5). During the thawing period, the changes in peak intensities were observed at resonances similar to freezing treatment (δ 0.8 to 1.6, 2.1 to 2.8, 3.2 to 3.9, and 5.2 to 5.5 ppm), but the peak intensity increases were much lower than during freezing treatment. In order to better understand the metabolite changes resulting from LPC treatment, 2D NMR experiments such as COSY, TOCSY, HSQC, and HMBC were run on samples from LPC, treated plants. The proton and carbon connectivity information revealed by these ^1^H-^1^H or ^1^H-^13^C correlation spectra indicated that the major metabolite changes are due to unsaturation of fatty acids (peaks at δ 0.8 to 1.6, 2.1 to 2.8, and 5.2 to 5.5 ppm) and sugar or sugar alcohols (peaks at δ 3.2 to 3.9, and 5.2 to 5.5 ppm) (Figure 6).

### Global transcriptional changes elicited by ANE

The transcript level changes during the post freezing recovery period were analyzed in ANE**-**treated Arabidopsis plants thawed at 4°C for 24h after a freezing treatment of −2°C for 24h (Additional file 1: Figure S1). LPC treatment affected the expression of about 5% (1113 genes) of the Arabidopsis genome (P≤0.05; change, ≥1.5 fold) when, exposed to −2°C for 24h (Additional file 1: Figure S1A). Using the gene expression data from control plants as the calibrator data set, we determined that about 2% (463 genes) of the differentially expressed genes were up-regulated and 3% (650 genes) were down-regulated during freezing stress. During post freezing recovery period, examination of the expression ratios of genes indicated that a relatively small portion of the genome was differentially expressed in plants treated with LPC as compared to controls. We found that 398 genes (1.65%) were differentially expressed more than 1.5 fold (P≤0.05) with 131 genes (0.54%) up-regulated and 267 genes (1.11%) genes down-regulated during post freezing recovery period (Additional file 1: Figure S1C).

### Functional classification

The genes that were differentially expressed during freezing or post**-**freezing recovery were classified into different functional categories, using the web-based tool Classification Super-Viewer, BLAST searches, and literature reports. The gene lists were classified according to the absolute gene number and normalized frequency. Genes associated with abiotic or biotic stimuli, transcription factor activity, response to stress, etc. ranked highest among the up-regulated genes (Additional file 1: Figure S2A), while those responsible for extra-cellular, cell wall, receptor binding, etc. ranked highest among down-regulated genes during freezing stress (Additional file 1: Figure S2C). During the post**-**freezing recovery period, functional classification of the differentially expressed genes showed an opposite trend to that of freezing, with extra-cellular, DNA-RNA metabolism, cell wall, etc. ranking highest in the up-regulated ones (Additional 1: Figure S3A) while response to abiotic and biotic stimuli, response to stress, cell wall, etc. in down-regulated ones (Additional file 1: Figure S3C).

When the genes were classified according to the actual number of genes which were differentially expressed, ‘unknown category’ (other molecular functions, other biological processes, other metabolic processes, other cellular processes, etc.) ranked at the top (Additional file 1: Figure S2B, S2D, S3B and S3D), indicating there are many genes altered by treatment with LPC during freezing stress and post**-**freezing recovery period. A list of selected differentially expressed genes (either up-regulated or down-regulated) and their functional categories are given in Tables 1 and 2.

**Table 1:** A list of selected up-regulated genes and their functional categories

**No**	**Locus**	**FC**	**Gene ontology class**	**Gene annotation detail**
**Response to stress and/or stress stimuli**				
1	At1g55920	2	response to cold	AtSerat2;1_SAT1_SAT5__AtSerat2;1 (SERINE ACETYLTRANSFERASE 1)
2	At3g22840	1.94	response to cold	ELIP1_ELIP__ELIP1 (EARLY LIGHT-INDUCABLE PROTEIN); chlorophyll binding
3	At1g10760	1.6	cold acclimation	SEX1_GWD_SOP__SEX1 (STARCH EXCESS 1)
4	At1g09780	1.5	response to cold	2,3-biphosphoglycerate-independent phosphoglyceratemutase, putative/phosphoglyceromutase, putative
5	At4g26080	1.73	response to heat	ABI1__ABI1 (ABA INSENSITIVE 1); calcium ion binding/protein serine/threonine phosphatase
6	At4g26850	1.61	response to heat	VTC2__VTC2 (VITAMIN C DEFECTIVE 2)
7	At1g73330	3.09	response to water deprivation	ATDR4__ATDR4 (Arabidopsis thaliana drought-repressed 4)
8	At3g11410	2.61	response to water deprivation	AHG3_ATPP2CA__AHG3/ATPP2CA (ARABIDOPSIS THALIANA PROTEIN PHOSPHATASE 2CA); protein binding/protein serine/threonine phosphatase
9	At5g62470	2.25	response to salt stress	AtMYB96_mybcov1_MYB96__MYB96 (myb domain protein 96); DNA binding/transcription factor
10	At3g61890	2.21	response to salt stress	ATHB-12_ATHB12__ATHB-12 (ARABIDOPSIS THALIANA HOMEOBOX PROTEIN 12); transcription factor
11	At2g46680	1.93	response to water deprivation	ATHB-7_ATHB7__ATHB-7 (ARABIDOPSIS THALIANA HOMEOBOX 7)
12	At5g57050	1.87	response to osmotic stress	ABI2__ABI2 (ABA INSENSITIVE 2)
13	At5g59780	1.84	response to salt stress	AtMYB59_MYB59__MYB59 (myb domain protein 59); DNA binding/transcription factor
14	At5g37500	1.82	response to water deprivation	GORK__GORK (GATED OUTWARDLY-RECTIFYING K+ CHANNEL); cyclic nucleotide binding/inward rectifier potassium channel/outward rectifier potassium channel
15	At4g34990	1.80	response to salt stress	AtMYB32__AtMYB32 (myb domain protein 32); DNA binding/transcription factor
16	At1g29395	1.76	response to water deprivation	COR414-TM1__COR414-TM1 (cold regulated 414 thylakoid membrane 1)
17	At1g02930	1.70	response to water deprivation	ATGSTF6_ATGSTF3_ERD11_GST1_GSTF6__ATGSTF6 (EARLY RESPONSIVE TO DEHYDRATION 11); glutathione transferase
18	At3g19580	1.68	hyperosmotic salinity response	AZF2__AZF2 (ARABIDOPSIS ZINC-FINGER PROTEIN 2)
19	At3g08720	1.67	response to salt stress	ATS6K2_S6K2_ATPK19_ATPK2__ATPK19 (ARABIDOPSIS THALIANA PROTEIN KINASE 19); kinase
20	At2g30870	1.65	response to water deprivation	ATGSTF10_ATGSTF4_ERD13__ATGSTF10 (EARLY DEHYDRATION-INDUCED 13); glutathione transferase
21	At4g30660	1.64	hyperosmotic salinity response	hydrophobic protein, putative/low temperature and salt responsive protein, putative
22	At3g55730	1.61	response to salt stress	AtMYB109_MYB109__MYB109 (myb domain protein 109); DNA binding/transcription factor
23	At3g03190	1.57	response to oxidative stress	ATGSTF11_ATGSTF6__ATGSTF11 (GLUTATHIONE S-TRANSFERASE F11); glutathione transferase
24	At5g01600	1.74	response to hydrogen peroxide	ATFER1__ATFER1 (FERRETIN 1); ferric iron binding
25	At2g16060	1.87	response to hypoxia	AHB1_ARATH GLB1_ATGLB1_GLB1_NSHB1__AHB1 (ARABIDOPSIS HEMOGLOBIN 1)
26	At1g05200	1.56	response to light stimulus	ATGLR3.4_GLR3.4_GLUR3__ATGLR3.4 (Arabidopsis thaliana glutamate receptor 3.4)
27	At5g64330	1.59	response to light stimulus	NPH3_JK218_RPT3__NPH3 (NON-PHOTOTROPIC HYPOCOTYL 3)
28	At1g21590	1.63	response to stress	protein kinase family protein
29	At2g35980	1.56	response to stress	YLS9_NHL10__YLS9 (YELLOW-LEAF-SPECIFIC GENE 9)
30	At2g38470	2.0	defense response to fungus	WRKY33__WRKY33 (WRKY DNA-binding protein 33); transcription factor
31	At1g12820	1.87	response to molecule of bacterial origin	AFB3__AFB3 (AUXIN SIGNALING F-BOX 3); auxin binding/ubiquitin-protein ligase
32	At2g19190	1.80	defense response to bacterium	FRK1__FRK1 (FLG22-INDUCED RECEPTOR-LIKE KINASE 1); kinase
33	At5g52450	1.73	response to nematode	MATE efflux protein-related
34	At4g12480	1.65	defense response to fungus	pEARLI 1__pEARLI 1; lipid binding
35	At1g24020	1.63	response to biotic stimulus	MLP423__MLP423 (MLP-LIKE PROTEIN 423)
36	At2g24570	1.63	defense response to bacterium	WRKY17__WRKY17 (WRKY DNA-binding protein 17); transcription factor
37	At5g64120	1.58	defense response to fungus	peroxidase, putative
38	At5g58600	1.55	defense response to fungus	PMR5__PMR5 (POWDERY MILDEW RESISTANT 5)
39	At3g11660	1.54	defense response to virus	NHL1__NHL1 (NDR1/HIN1-like 1)
40	At5g50200	2.53	response to wounding	ATNRT3.1_NRT3.1_WR3__WR3 (WOUND-RESPONSIVE 3); nitrate transmembrane transporter
41	At5g13930	1.51	response to wounding	CHS_TT4__ATCHS/CHS/TT4 (CHALCONE SYNTHASE); naringenin-chalcone synthase
**Sugar Accumulation**				
1	At1g56600	3.19	carbohydrate biosynthetic process	ATGOLS2 (ARABIDOPSIS THALIANA GALACTINOL SYNTHASE 2); transferase, transferring glycosyl groups/transferase, transferring hexosyl groups
2	At5g18470	2.63	sugar binding	curculin-like (mannose-binding) lectin family protein
3	At1g02460	2.46	carbohydrate metabolic process	glycoside hydrolase family 28 protein/polygalacturonase (pectinase) family protein
4	At3g46970	1.85	carbohydrate metabolic process	ATPHS2/PHS2 (ALPHA-GLUCAN PHOSPHORYLASE 2); phosphorylase/transferase, transferring glycosyl groups
5	At4g19810	1.84	carbohydrate metabolic process	glycosyl hydrolase family 18 protein
6	At1g48100	1.84	carbohydrate metabolic process	glycoside hydrolase family 28 protein/polygalacturonase (pectinase) family protein
7	At5g41670	1.79	pentose-phosphate shunt	6-phosphogluconate dehydrogenase family protein
8	At5g66460	1.75	carbohydrate metabolic process	(1–4)-beta-mannanendohydrolase, putative
9	At3g18080	1.72	carbohydrate metabolic process	glycosyl hydrolase family 1 protein
10	At2g44450	1.68	carbohydrate metabolic process	glycosyl hydrolase family 1 protein
11	At4g27830	1.62	carbohydrate metabolic process	glycosyl hydrolase family 1 protein
12	At4g09020	1.61	starch catabolic process; alpha-amylase activity	ATISA3_ISA3__ATISA3/ISA3 (ISOAMYLASE 3); alpha-amylase
13	At3g61490	1.61	carbohydrate metabolic process	glycoside hydrolase family 28 protein/polygalacturonase (pectinase) family protein
14	At1g10760	1.58	starch catabolic process	SEX1_GWD_SOP__SEX1 (STARCH EXCESS 1)
15	At4g15210	1.54	starch catabolic process	BMY1_ATBETA-AMY_AT-BETA-AMY_RAM1__ATBETA-AMY (BETA-AMYLASE); beta-amylase
16	At2g40840	1.5	starch catabolic process	DPE2__DPE2 (DISPROPORTION ATING ENZYME 2); 4-alpha-glucanotransferase/heteroglycan binding
17	At1g03310	1.5	starch metabolic process	ATISA2/BE2/DBE1/ISA2 (DEBRANCHING ENZYME 1); alpha-amylase/isoamylase
**Lipid metabolism**				
1	At4g39670	4.88	glycolipid binding; molecular function unknown	glycolipid binding/glycolipid transporter
2	At2g37870	2.25	lipid binding; lipid transport	protease inhibitor/seed storage/lipid transfer protein (LTP) family protein
3	At2g24560	2.04	lipid metabolic process	carboxylesterase
4	At3g22120	2.01	lipid transport; lipid binding	CWLP (CELL WALL-PLASMA MEMBRANE LINKER PROTEIN); lipid binding
5	At5g55450	1.98	lipid transport; lipid binding	protease inhibitor/seed storage/lipid transfer protein (LTP) family protein
6	At4g10955	1.75	lipid metabolic process	lipase class 3 family protein
7	At4g12500	1.72	lipid binding	protease inhibitor/seed storage/lipid transfer protein (LTP) family protein
8	At5g64080	1.71	lipid binding	protease inhibitor/seed storage/lipid transfer protein (LTP) family protein
9	At2g38530	1.61	phospholipid transfer to membrane	LTP2 (LIPID TRANSFER PROTEIN 2); lipid binding
**ABA Responsive**				
1	At1g63840	3.27	Response to ABA stimulus; endome- mbrane system	Zinc finger (C3HC4-type RING finger) family protein
2	At5g01540	3.22	response to abscisic acid stimulus	lectin protein kinase, putative
3	At3g61890	2.21	response to abscisic acid stimulus	ATHB-12 (ARABIDOPSIS THALIANA HOMEOBOX PROTEIN 12); transcription factor
4	At1g69270	2.04	abscisic acid mediated signaling	RPK1 (RECEPTOR-LIKE PROTEIN KINASE 1); kinase
**Others**				
1	At1g21120	3.96	O-methyltransferase activity	O-methyltransferase, putative
2	At1g78410	3.79	Response to oxidative stress; Molecular function unknown	VQ motif-containing protein
3	At2g21210	3.31	Molecular function unknown	auxin-responsive protein, putative
4	At5g06760	3.18	Molecular function unknown	late embryogenesis abundant group 1 domain-containing protein/LEA group 1 domain-containing protein
5	At1g73330	3.09	response to water deprivation	ATDR4__ATDR4 (Arabidopsis thaliana drought-repressed 4)
6	At1g24140	2.67	anchored to membrane	matrixin family protein
7	At3g11410	2.61	response to water deprivation	AHG3_ATPP2CA__AHG3/ATPP2CA (ARABIDOPSIS THALIANA PROTEIN PHOSPHATASE 2CA); protein binding/protein serine/threonine phosphatase
8	At2g15390	2.05	cell wall biogenesis; Fucosyltransferase activity	FUT4 (fucosyltransferase 4); fucosyltransferase
9	At1g55920	1.97	response to cold	AtSerat2;1_SAT1_SAT5__AtSerat2;1 (SERINE ACETYLTRANSFERASE 1)
10	At3g22840	1.94	response to cold	ELIP1_ELIP__ELIP1 (EARLY LIGHT-INDUCABLE PROTEIN); chlorophyll binding
11	At3g55130	1.84	ATPase activity, coupled to transmembrane movement of substances	ATWBC19 (WHITE-BROWN COMPLEX HOMOLOG 19); ATPase, coupled to transmembrane movement of substances
12	At4g26850	1.61	L-ascorbic acid biosynthetic process	VTC2 (VITAMIN C DEFECTIVE 2)

**Table 2:** A list of selected down-regulated genes and their functional categories

**No**	**Locus**	**FC**	**Gene ontology class**	**Gene annotation detail**
**Carbohydrate adjustments**				
1	At5g49360	3.07	hydrolase activity, hydrolyzing O-glycosyl compounds	BXL1__BXL1 (BETA-XYLOSIDASE 1); hydrolase, hydrolyzing O-glycosyl compounds
2	At2g19800	2.93	inositol oxygenase activity	MIOX2__MIOX2 (MYO-INOSITOL OXYGENASE 2)
3	At4g30280	2.82	glucan metabolic process	ATXTH18_XTH18__ATXTH18/XTH18 (XYLOGLUCAN ENDOTRANSGLUCOSYLASE/HYDROLASE 18); hydrolase, acting on glycosyl bonds
4	At5g52050	2.49	drug transporter activity	MATE efflux protein-related
5	At1g02640	2.49	hydrolase activity, hydrolyzing O-glycosyl compounds	BXL2__BXL2 (BETA-XYLOSIDASE 2); hydrolase, hydrolyzing O-glycosyl compounds
6	At1g23480	2.45	transferase activity, transferring glycosyl groups	ATCSLA03_ATCSLA3_CSLA03__ATCSLA03 (Cellulose synthase-like A3); transferase, transferring glycosyl groups
7	At3g27540	2.42	transferase activity, transferring glycosyl groups	glycosyltransferase family 17 protein
8	At3g06770	2.36	polygalacturonase activity	glycoside hydrolase family 28 protein/polygalacturonase (pectinase) family protein
9	At1g10550	2.31	glucan metabolic process	XET_XTH33__XTH33 (xyloglucan:xyloglucosyltransferase 33); hydrolase, acting on glycosyl bonds
10	At1g10400	2.29	transferase activity, transferring glycosyl groups	UDP-glycosyltransferase/transferase, transferring glycosyl groups
11	At4g25810	2.24	glucan metabolic process	XTR6_XTH23__XTR6 (XYLOGLUCAN ENDOTRANSGLYCOSYLASE 6); hydrolase, acting on glycosyl bonds
12	At1g23870	2.17	trehalose-phosphatase activity	ATTPS9_TPS9__ATTPS9 (Arabidopsis thaliana trehalose-phosphatase/synthase 9); transferase, transferring glycosyl groups/trehalose-phosphatase
13	At1g12240	2.12	sucrose catabolic process, using beta-fructofuranosidase	ATBETAFRUCT4_VAC-INV__ATBETAFRUCT4/VAC-INV (VACUOLAR INVERTASE); beta-fructofuranosidase/hydrolase, hydrolyzing O-glycosyl compounds
14	At5g56870	2.09	beta-galactosidase activity	BGAL4__BGAL4 (beta-galactosidase 4); beta-galactosidase
15	At2g47930	2.06	anchored to membrane	AGP26__AGP26/ATAGP26 (ARABINOGALACTAN PROTEINS 26)
16	At4g30290	2.05	glucan metabolic process	ATXTH19__ATXTH19 (XYLOGLUCAN ENDOTRANSGLUCOSYLASE/HYDROLASE 19); hydrolase, acting on glycosyl bonds
17	At5g27350	2.04	sugar porter activity	SFP1__SFP1; carbohydrate transmembrane transporter/sugar:hydrogen ion symporter
18	At2g32540	2.02	polysaccharide biosynthetic process	ATCSLB04_ATCSLB4_CSLB04__ATCSLB04 (Cellulose synthase-like B4); transferase/transferase, transferring glycosyl groups
19	At3g62720	1.86	polysaccharide biosynthetic process	ATXT1__ATXT1; transferase/transferase, transferring glycosyl groups
20	At4g37800	1.77	glucan metabolic process	xyloglucan:xyloglucosyltransferase, putative/xyloglucanendotransglycosylase, putative/endo-xyloglucantransferase, putative
21	At1g11260	1.65	sugar porter activity	STP1__STP1 (SUGAR TRANSPORTER 1); carbohydrate transmembrane transporter/sugar:hydrogen ion symporter
22	At1g75220	1.5	sugar porter activity	integral membrane protein, putative
**Lipid metabolism**				
1	At4g22753	2.84	catalytic activity	SMO1-3_ATSMO1_SMO1__SMO1-3 (STEROL 4-ALPHA METHYL OXIDASE); catalytic
2	At5g52050	2.49	drug transporter activity	MATE efflux protein-related
3	At1g17420	2.37	lipoxygenase activity	LOX3__LOX3 (Lipoxygenase 3); iron ion binding/lipoxygenase/metal ion binding/oxidoreductase, acting on single donors with incorporation of molecular oxygen, incorporation of two atoms of oxygen
4	At3g47560	2.27	catalytic activity	esterase/lipase/thioesterase family protein
5	At5g24150	2.22	sterol biosynthetic process	SQP1__SQP1 (Squalenemonooxygenase 1)
6	At3g23470	2.1	lipid biosynthetic process	cyclopropane-fatty-acyl-phospholipid synthase
7	At1g28600	1.94	lipid metabolic process	lipase, putative
8	At5g18630	1.93	lipid metabolic process	lipase class 3 family protein
9	At5g24210	1.92	lipid metabolic process	lipase class 3 family protein
10	At1g02660	1.79	lipid metabolic process	lipase class 3 family protein
11	At3g23510	1.77	lipid biosynthetic process	cyclopropane fatty acid synthase, putative/CPA-FA synthase, putative
12	At4g26790	1.73	lipid metabolic process	GDSL-motif lipase/hydrolase family protein
13	At2g42690	1.68	lipid metabolic process	lipase, putative
14	At1g28670	1.67	lipid metabolic process	ARAB-1__ARAB-1 (Arabidopsis lipase); carboxylesterase
15	At4g13050	1.55	fatty acid biosynthetic process	acyl-(acyl carrier protein) thioesterase, putative/acyl-ACP thioesterase, putative/oleoyl-(acyl-carrier protein) hydrolase, putative/S-acyl fatty acid synthase thioesterase, putative
16	At1g64400	1.5	fatty acid biosynthetic process	long-chain-fatty-acid--CoA ligase, putative/long-chain acyl-CoA synthetase, putative
**Others**				
1	At1g12610	6.03	gibberellin biosynthetic process	DDF1__DDF1 (DWARF AND DELAYED FLOWERING 1); DNA binding/transcription factor
2	At2g37180	2.36	membrane	RD28_PIP2;3_PIP2C__RD28 (plasma membrane intrinsic protein 2;3); water channel
3	At3g30775	2.22	proline catabolic process	PRODH_ERD5_ATPDH_ATPOX_AT-POX_PRO1__ERD5 (EARLY RESPONSIVE TO DEHYDRATION 5); proline dehydrogenase
4	At4g19420	2.03	carboxylic ester hydrolase activity	pectinacetylesterase family protein
5	At4g22200	1.95	regulation of membrane potential	AKT2/3_AKT2_AKT3__AKT2 (Arabidopsis K+ transporter 2); cyclic nucleotide binding/inward rectifier potassium channel
6	At5g21100	1.92	endomembrane system	L-ascorbate oxidase, putative
7	At1g19670	1.59	chlorophyllase activity	ATCLH1_ATHCOR1_CORI1__ATCLH1 (CORONATINE-INDUCED PROTEIN 1)

### Pathway analysis

#### LPC**-**mediated differential gene expression in Arabdopsis

**Figure 7:**
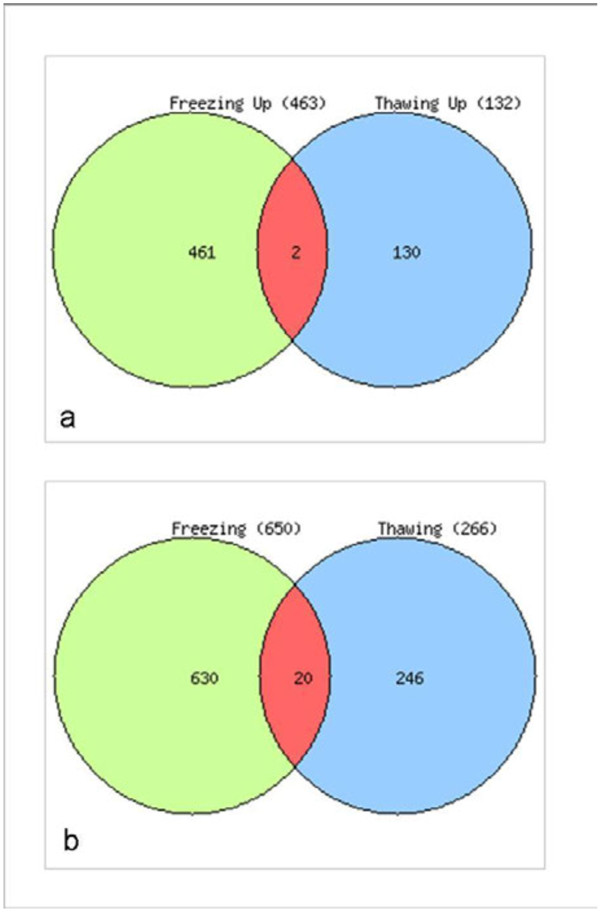
**Diagram of representative metabolic pathways regulated by the LPC treatment during freezing stress.** Each pathway is shown as a glyph consisting of nodes and lines, which represent the metabolites and reactions, respectively. Expression-level change of each reaction is shown in a color relative to the expression level, as indicated in the color scale bar. Triangle, amino acids; square, carbohydrates; diamond, proteins; open circle, others; closed circle, phosphorylated.

**Figure 8:**
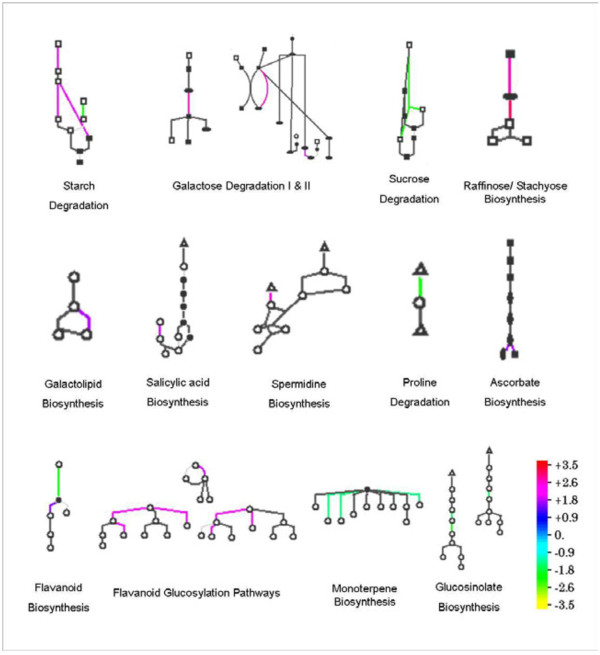
Venn diagram showing the number of significantly (a) up or (b) down-regulated and the shared genes during freezing stress and post-freezing recovery period.

Standardized AraCyc-defined metabolic pathways were used to study the metabolic changes in LPC**-**treated Arabidopsis plants during freezing and post**-**freezing recovery. To examine the similarities and differences in the concerned metabolic pathways during the freezing stress and post**-**freezing recovery periods, entire biosynthetic pathways of Arabidopsis were analyzed. During freezing, out of the total 1113 genes which were differentially expressed (either up-regulated or down-regulated), 334 genes were identified by the Pathway Tools Omics Viewer, and of these 179 genes were assigned specific pathway. On the other hand, 138 genes out of total 398 genes were identified and 66 genes were assigned pathway during the post freezing recovery period. We found that specific pathways were affected significantly by LPC treatment, with little overlap during freezing and post**-**freezing recovery (Figure 7). The major known pathways found to be affected were soluble sugar accumulation, lipid metabolism, hormonal balance, flavanoid/terpene biosynthesis, proline degradation, and energy homeostasis (Figure 8).

Major changes observed were the multi-step promotion of the starch degradation pathway (SEX1, SEX4, AT1G03310, AT4G09020, AT4G15210, AT3G46970), inactivation of sucrose degradation (AT1G12240.2.12), induction of galactose degradation pathways I & II (MUR4, AT2G34850), stimulation of raffinose/stachyose biosynthesis (UGE2, UGE5, GolS2, GolS3), and down-regulation of trehalose synthesis (TPS8, TPS9), and gluconeogenesis (AT1G09780) (Figure 8).

A major pathway involved in galactolipid biosynthesis and lipid trafficking in thylakoid membranes (DGD1) was specifically affected (Figure 8). Other changes in lipid metabolism included down-regulation of sterol/cholesterol biosynthesis (AT5G24150), and down regulation of long chain fatty acid synthesis (AT1G01120). Additionally, the salicylic acid biosynthesis pathway was found to be stimulated in ANE**-**treated plants upon exposure to freezing stress. AT1G18870, which encodes a protein with isochorismate synthase activity, was found to be activated during freezing stress (Figure 8). Mutant studies of this gene's function suggest that its function is redundant with that of ICS1 (AT1G7410). Moreover, a UDP-glycosyltransferase (AT2G43820) which is induced by salicylic acid during biotic stress, was also found to be activated during this period. On the other hand, the isochorismate synthase gene, ICS1 (AT1G7410) was found to be specifically down-regulated during post**-**freezing recovery.

During freezing, the hormonal balance pathway responsible for conjugation of cytokinins involving *N*-glucosylation was highly induced while the same pathway was specifically down-regulated during the post**-**freezing recovery period. UDP-glycosyltransferases like UGT73B2, UGT76C2, UGT76C1, AT2G43820, and At1G24100 were highly affected by the treatment. Down-regulation of the gibberellin inactivation pathway was also observed, with GA2-oxidase (GA2ox1), a dioxygenase involved in GA inactivation being specifically down-regulated.

A polyamine biosynthetic gene (AT5G15950) involved in spermine/spermidine biosynthesis was up-regulated in LPC treated plants. Pathway analysis of proline dehydrogenase (*prodh*), the gene involved in degradation of proline, showed that application of LPC down-regulated the gene thereby maintaining a higher level of proline throughout the freezing and post-freezing recovery periods. The ascorbate biosynthetic gene (VTC2), involved in the response to jasmonic acid stimulus (Figure 8), and L-aspartate oxidase (At5g14760), involved in the early steps of NAD biosynthesis, were both up-regulated. Thus, the free radical scavenging pathway was also found to be activated. Furthermore, genes responsible for flavanoid biosynthesis (AT5G13930, 4CL1) and glucosylation (UGT73B2) were up-regulated, while monoterpene (TPSO3) and glucosynalate (At1G24100) biosynthetic genes were down-regulated by freezing stress in plants treated with LPC (Figure 8).

#### Specificity of LPC treatment at the transcriptional level

A rather limited gene overlap was found between the ANE treatment and the control, as shown in the Venn diagram (Figure 7). When the gene lists were mined for common candidates during freezing and thawing, there were only two and 20 genes in common in the up- and down-regulated classes, respectively. A list of common genes which showed a similar pattern of expression (either up-regulated or down-regulated) during freezing and post**-**freeze thawing are given in Additional file 2: Table S2.

#### Heat map

To get a broad overview of the expression pattern of individual genes during freezing and post**-**freezing recovery, a heat map was generated using the web-based tool, Heat Mapper Plus. A representative sample of the heat map of the global changes in the transcript levels of genes during freezing and the post**-**freezing recovery period is given in Additional file 1: Figure S4.

#### Verification of microarray data by quantitative RT–PCR

Several transcripts of selected up-regulated genes based on microarray analysis were quantified independently by RT–PCR (Additional file 2: Table S3). They include a glycolipid transportor (At4g39670), VQ motif-containing protein (At1g78410), ABA responsive zinc finger family protein (At1g63840), galactinol synthase (At1g56600), a drought-repressed protein (At1g73330), the cell wall protein precursor (At2g20870), an expansin protein (At2g40610), a gibberellin-responsive protein (At1g74670), the lipid transfer protein (LTP) (At4g22490), and an invertase/pectin methylesterase inhibitor (At5g20740). The RT–PCR results of all 10 genes showed similar differential expression patterns to those obtained by microarray analysis (Data not shown).

## Discussion

The results presented in this paper show that lipophilic components of *A. nodosum* extracts (LPC) induced specific biochemical changes leading to enhanced tolerance to freezing stress in *Arabidopsis thaliana*. LPC-treatment of the roots resulted in enhanced freezing tolerance in the aerial parts of the plant, which implicates LPC in inducing systemic metabolic responses.

Proline is one of several compounds with roles as compatible solutes that accumulate in response to freezing and drought stress, [33] and osmotic stress [16]. Our results on the quantification of proline accumulation in wild-type and LPC treated Arabidopsis plants showed that application of ANE or LPC, resulted in a 50% increase in free proline in response to freezing, as compared to control plants.

In higher plants, proline is synthesized via the glutamate pathway or the ornithine pathway [18,22,33]. The glutamate pathway is predominant during abiotic stress and under nitrogen limiting conditions [33,34], while the ornithine pathway takes effect under nitrogen abundance [33]. Plants accumulate proline in response to freezing-induced osmotic stress by simultaneous activation of proline biosynthesis (via the *P5CS1* pathway) and/or down regulation of proline degradation (via the *ProDH* pathway) [35]. The reciprocal regulation of the Δ^1^-pyrroline-5-carboxylate synthetase (P5CS), a rate-limiting enzyme in proline biosynthesis, and proline dehydrogenase (*ProDH*), responsible for proline degradation, is a key mechanism in the control of cytosolic proline concentration [34].

The results of our experiments with *p5cs1* mutant plants confirmed the role of proline in ANE-mediated freezing tolerance, in that the *p5cs1* mutants, treated with ANE, LPC and the water control, all showed similar freezing damage. These observations were in agreement with the results of the gene expression studies. Results of the gene expression analysis suggested that increased proline concentration in the LPC**-**treated plants was a result of a coordinated increase in *P5CS1* and *P5CS2* transcripts and, in part, due to decreased in *ProDH*.

Several studies have suggested that cold-induced sugar accumulation enhances the degree of plant freezing tolerance. In Arabidopsis, a large increase in the degree of freezing tolerance is positively correlated with soluble sugar content [36,37]. Similarly, increased sucrose levels in transgenic Arabidopsis plants, over-expressing a gene for sucrose phosphate synthase, equated with freezing tolerance [38]. Conversely, sensitive to freezing 4 (*sfr4*) mutants exhibited an impaired freezing tolerance response due to a reduced accumulation of sugar levels at low temperature relative to the wild type [39,40]. In LPC**-**treated plants, increased accumulation of soluble carbohydrates was brought about by multiple mechanisms such as inducing polysaccharide degradation (i.e. starch, galactose etc.), promoting the biosynthesis of soluble carbohydrates (i.e. glucose, fructose, Raffinose/stachyose), and inactivating the sucrose degradation pathways.

Recently, Uemura et al. [40] showed that the sensitivity of the Arabidopsis mutant *sfr4* to freezing was due to its low sugar content, as manifested by loss of osmotic responsiveness. Additionally, it has been demonstrated that exogenous sucrose, at low concentrations, serves as a substrate for low temperature-induced metabolic alterations, while at higher concentrations it has a direct cryoprotective effect on cellular membranes [41]. Although an increased concentration of cytosolic soluble sugar has been observed in response to freezing-induced osmotic stress, in many plant species [36], it is unclear whether the accumulated sugars act as osmolytes, or serve as a source of energy and carbon, that fuels the metabolic changes leading to enhanced freezing tolerance [42].

A positive correlation between sugar accumulation and freezing tolerance has been widely documented in many plant species, including Arabidopsis [36,43]. In the present study, application of ANE or LPC resulted in a significantly enhanced accumulation of total soluble sugars in response to freezing, as compared to untreated controls. The results were further strengthened by analyzing the sensitivity of *sfr4* mutant plants to freezing.

^1^H NMR analysis of the Arabidopsis metabolome, during freezing, revealed that the application of LPC altered biochemical pathways resulting in the accumulation of specific metabolites, leading to enhanced freezing tolerance. The ^1^H NMR spectra of plants treated with LPC was dominated by peaks with chemical shifts (δ) around 0.8 to 4.0 ppm. These chemical shifts primarily represented 2 major groups of compounds; 0.8 – 2.8 ppm represents lipophilic components like fatty acids and sterols, while resonance at 3.0 to 4.0 ppm represented carbohydrates, sugars, sugar alcohols and organic acids [44]. Our results are in agreement with previous reports. These findings showed that multiple primary metabolites could act collectively, as compatible solutes, ameliorating the osmotic stress caused by freezing.

^1^H NMR peaks with chemical shifts (δ) around 0.8 to 2.8 ppm, represented the lipophilic components such as fatty acids and sterols, were another major group of metabolites which showed specific changes during freezing stress in LPC**-**treated plants. Fatty acids are major components of cellular membranes, suberin, and cutin waxes that provide structural barriers to the environment. At low temperature, plant membranes undergo a transition from a liquid crystalline state to a gel-like phase with reduced fluidity contributing to ion leakage and deactivation of membrane proteins [44]. Fatty acids contribute to inducible stress resistance through the remodeling of membrane fluidity [45]. The ability to adjust membrane fluidity by modulating levels of unsaturated fatty acids is a feature of freezing-tolerant plants. Fatty acid unsaturation is thought to reduce the propensity of cellular membranes to undergo freezing-induced, non-bilayer phase formation, thus enhancing membrane integrity and cellular function during freezing [44].

Metabolite profiling revealed an increase in unsaturated fatty acids in plants treated with LPC when exposed to freezing. The spectral peaks (or peak groups) at δ = 5.4-5.2 ppm (protons on double bond carbons), 2.8, 2.3, 2.1, 1.6, 1.4-1.2 ppm (protons on alkyl chain), and 1.0-0.8 ppm (terminal methyl group of alkyl chain) are indicative of unsaturation of fatty acid. We have previously reported that application of ANE or LPC resulted in less tissue damage and electrolyte leakage, as compared to controls, thereby improving the LT_50_ values [24]. The metabolite profiles suggested that that LPC-mediated freezing tolerance in Arabidopsis may be the result of a combination of metabolic adjustments and increased fatty acid content in Arabidopsis.

Chemical components in the extracts of *A. nodosum* caused a rapid biochemical response leading to an increased accumulation of osmoprotectants (proline and soluble sugars) and unsaturated fatty acids. The results of the present study suggested that application of these extracts elicited responses reminiscent of a priming effect. Priming is a phenomenon whereby previous exposure to biotic or abiotic stress stimuli makes a plant more resistant to future incidents [46]. Chemical priming is a novel strategy, wherein application of certain chemicals mimic moderate stress stimuli, through physiological and/or hormonal reactions and brings about a priming reaction in the target plants. Exogenous application of chemicals inducing a priming effect, in abiotic stress tolerance has been previously reported [47]. Application of a non-protein amino acid, β-aminobutyric acid (BABA), was shown to induce a priming response in Arabidopsis towards salt stress tolerance (through induction of ABA**-**dependant elements such as *RAB18*and *RD29A*) and salicylic acid**-**dependent disease tolerance (through *PR-1*, *PR-5* response) [47]. The results presented in this paper suggest the possibility of a chemical priming effect in Arabidopsis, as a reaction to the application of ANE or LPC, resulting in enhanced freezing tolerance.

Further, we used a whole-genome approach to determine the ANE**-**mediated freezing tolerance in Arabidopsis plants. Application of LPC significantly altered gene expression for 1% of the Arabidopsis genes. In this study, we focused on gene expression related to three aspects: (1) response to stress and/or stress stimulus; (2) compatible osmolyte accumulation; (3) changes in membrane lipid profile in response to stress.

Around 40 annotated genes were found to be up-regulated in our focus category with four genes (At1g10760, At1g55920, At1g09780, At3g22840) found to be directly involved in low temperature stress tolerance in Arabidopsis [48-51]. Another major group of induced genes was those expressed in response to osmotic stress (like water deprivation, salt stress etc.). The ability to survive periods of desiccation is an important adaptation to freezing temperatures and many of the ‘water-deprivation controlled’ genes are also required for maximum survival during freezing stress [52]. Many of the genes expressed during cold acclimation are also inducible by drought stress, and are likely to play a role in protection against cellular dehydration, which occurs during both freezing and drought conditions [53]. This finding is consistent with previous observations, that those plants which are able to efficiently manage osmotic stress, are more tolerant to freezing temperatures [54].

Genes activated in response to biotic stress and/or stimuli were also activated owing to the fact that both biotic and abiotic stress stimuli share a common signaling cascade to bring about physiological responses [55]. For example, salicylic acid (SA) has long been known as a signal molecule in the induction of defense mechanisms in plants [56-58]. Exogenous application of SA has been shown to improve freezing tolerance in wheat by regulating the ice nucleation activity of apoplastic proteins [59,60]. Ding et al. [61] have shown that 0.01 mM methyl salicylate and methyl jasmonate treatment improved the cold tolerance of tomato fruits. Moreover, SA was shown to accumulate during low temperatures in chilling-resistant Arabidopsis plants [62].

Similarly, several studies have suggested that cold-induced sugar accumulation enhances the degree of plant freezing tolerance. In Arabidopsis, a large increase in the degree of freezing tolerance is positively correlated with soluble sugar content [36,37]. Increased sucrose levels in transgenic Arabidopsis plants over-expressing a gene for sucrose phosphate synthase equated with freezing tolerance [38]. Conversely, sensitive to freezing 4 (*sfr4*) mutants exhibited an impaired freezing tolerance response due to a reduced accumulation of sugar levels at low temperature, relative to the wild type [39,40]. In LPC**-**treated plants, increased accumulation of soluble carbohydrates was brought about by multiple mechanisms such as induction of polysaccharide degradation (i.e. starch, galactose etc.), promoting biosynthesis of soluble carbohydrates (i.e. glucose, fructose, raffinose/stachyose) and inactivating the sucrose degradation pathways.

Starch is the main carbohydrate store in plants. Regulation of starch metabolism, in particular in response to environmental cues, is of primary importance for carbon and energy flow in plants [63]. Along with photosynthesis, starch degradation also plays a significant role in cold-induced sugar accumulation and enhanced freezing tolerance in Arabidopsis [43,48,64]. Starch-related glucan/water dikinases encoded by the Arabidopsis ‘Starch Excess’ 1 and 4 genes (SEX1, SEX4), regulate starch degradation in plastids by phosphorylating starch, thereby ensuring better accessibility by starch-degrading enzymes during cold induced starch degradation. SEX1 plays an essential role in the cold-induced starch degradation, sugar accumulation, and freezing tolerance enhancement during an early phase of cold acclimation [48]. SEX4/DSP4 phosphatase activity has been shown to be regulated by the redox state [65] and unfavourable environmental stress conditions alter the redox balance within the cells. It is believed that SEX4/DSP4 phosphatase activity may be regulated in response to environmental stress and might be associated to MsK4/AtK-1, a plastid-localized protein kinase associated with starch granules, which is an important regulator that adjusts carbohydrate metabolism during environmental stress [63]. SEX 1 and SEX 4 were found to be activated in LPC**-**treated plants during freezing stress, as compared to control plants.

Raffinose family oligosaccharides (RFOs) such as raffinose and stachyose are accumulated during the process of cold acclimation, when plants acquire increased frost tolerance [66-68]. Arabidopsis plants with higher rates of raffinose biosynthesis demonstrated increased accumulation of raffinose and galactinol upon cold acclimation and exhibited higher freezing tolerance [67]. Molecular mechanisms by which RFOs influence cellular freezing tolerance are not clear, but it has been shown previously that raffinose can stabilize isolated chloroplast thylakoid membranes during a freeze–thaw cycle [69]. Galactinol synthase (GolS) catalyses the first committed step in the biosynthesis of RFOs [70] and, therefore its expression, provides an experimental tool to assess the level of RFOs during freezing stress, to analyze the function of RFOs as osmoprotectants [67]. LPC application stimulated the raffinose/stachyose biosynthetic genes GolS2 and GolS3 during freezing.

LPC treatment specifically induced a major pathway involved in galactolipid biosynthesis and lipid trafficking in thylakoid membranes controlled by the gene DGD1. Other changes include down-regulation of sterol/cholesterol biosynthesis, regulation of Acyl-CoA thioesterases and down regulation of long chain fatty acid synthesis (Figure 8).

Membranes are the major injury sites during freezing stress [71,72], and membrane lipids undergo substantial changes when plants are exposed to freezing temperatures [73]. It has been well established that membrane polar lipid composition is one of the important factors controlling the structure and efficiency of thylakoid membranes via specific lipid–protein interactions and/or the dynamic properties of the lipid bilayer [74,75]. The galactolipids constitute the bulk (close to 80%) of the thylakoid lipid matrix and, within green plant parts, 70 to 80% of the lipids are associated with photosynthetic membranes. During freezing, dramatic alterations take place in plastid membranes, decreasing the monogalactosyldiacylglycerol (MGDG) content and increasing digalactosyldiacylglycerol (DGDG) content [76,77]. The ratio of DGDG to MGDG is critical for correct protein folding, insertion and intracellular protein trafficking in the chloroplast [78] during temperature stress [79]. DGD1 (digalactosyldiacylglycerol synthase 1) is the enzyme involved in the conversion of MGDG to DGDG in photosynthetic membranes of the chloroplast [80]. The *dgd1* mutant of Arabidopsis was impaired in galactolipid assembly, as suggested by a 90% reduction in digalactosyl lipid content [80]. DGD1 (digalactosyldiacylglycerol synthase 1) was found to be highly expressed in LPC**-**treated plants during freezing stress. Taken together, it may be concluded that activation of digalactosyldiacylglycerol synthase 1 (DGD1) resulted in efficient conversion of MGDG to DGDG, thereby maintaining improved stability of membranes and reduced ion leakage in LPC treated plants.

An early response to low temperature stimulus, by tolerant plant species like winter barley oats, is decreased in membrane fluidity [71]. A major adaptation among the winter tolerant species is the ability to maintain the fluidity of membranes by reducing the ratio of free sterol to total phospholipids. This is achieved by a decrease in the free sterols and an increase in the proportion of phospholipids [81]. Sterol/cholesterol biosynthesis was found to be highly down regulated in LPC**-**treated plants during freezing stress. A further modification observed in the lipid metabolism of LPC treated plants was the regulation of Acyl-CoA thioesterase.

## Conclusions

We found that lipophilic components of ANE (LPC) modulated specific metabolic pathways in *A. thaliana* resulting in enhanced tolerance to freezing stress. Response to stress and/or stress stimuli, accumulation of compatible osmoprotectants, and regulation of the membrane lipid profile, were the major adaptations observed in LPC**-**treated plants during freezing stress. There was little overlap in the genes involved in tolerance to freezing stress versus post freezing recovery, indicating the specificity of the mode of action of LPC during freezing stress. A number of genes in the ‘unknown category’ (other molecular functions, other biological processes, other metabolic processes, other cellular processes, etc.) were differentially expressed, indicating that a number of potential genes had been altered by LPC treatment during freezing stress and post**-**f reezing recovery period. Further studies are needed to dissect the specific genes identified in this study**-**involved in specific pathways of stress signaling/response, osmolyte accumulation, and lipid metabolism and their roles in *A. nodosum* extract**-**mediated, enhanced freezing tolerance in *A. thaliana*.

## Methods

### Plant material and *Ascophyllum nodosum* extract

Seeds of *Arabidopsis thaliana* (col-0) were purchased from Lehle Seeds (Round Rock, TX). Murashige and Skoog [82] basal salt mixture (Cat No: M5524), sucrose and agar, were purchased from Sigma Aldrich (Oakville, ON).

### Preparation of *A. nodosum* extract (ANE) and its lipophilic fraction (LPC)

A commercial formulation of powdered, alkaline extract of *A. nodosum* was provided by Acadian Seaplants Limited, Dartmouth, Nova Scotia, Canada. An aqueous solution of the commercial formulation of *A. nodosum* extract (hereafter referred to as ANE) was prepared by dissolving 1 g of the extract powder in 20 ml of sterile distilled water by constant stirring for 15 min. The solution was then sterilized using a 0.22 μm filter (Corning Inc. NY, USA) and stored in sterile glass centrifuge tubes at 4°C until further use.

The lipophilic fraction (hereafter referred to as LPC) was prepared from a methanol extract of ANE by sequential fractionation with hexane, chloroform and ethyl acetate as described previously by Rayirath et al. [83]. The ethyl acetate sub-fraction, rich in lipophilic components such as fatty acids and sterols, was dried under a stream of nitrogen and stored in sterile glass centrifuge tubes at −80°C until further use. This fraction, re-suspended in a minimal quantity of methanol (100 μl/g equivalent) and diluted with sterile distilled water, was used as LPC for further experiments.

### Phenotypic studies and sample collection

#### Petri dish freezing tolerance assay

The *in vitro* Petri dish freezing tolerance assay, used to investigate the plant responses in ANE-induced freezing tolerance, was conducted as described by Xin and Browse (1998). Briefly, sterilized *Arabidopsis* seeds were planted on solidified Murashige and Skoog basal salt medium (Murashige and Skoog 1962), containing 1% sucrose and supplemented with different concentrations of ANE or LPC. Required concentrations of filter**-**sterilized ANE (0.5 gL^-1^) or LPC (1.0 gL^-1^ equivalent of ANE) were added to molten (50°C) media, and plated in partitioned petri dishes (Fisher Scientific, Ottawa, ON). Two sets of controls, one set of plates with distilled water used to dissolve ANE, and a second set with equal amount of methanol used to dissolve LPC (100 μl L^-1^ medium) were maintained. Seeds were evenly distributed in the Petri dishes by placing individual seeds with a 100 μL micro pipette at the rate of 10–15 seeds per partition. The petri dishes were incubated at 22/18°C day/night temperatures, 16:8 photoperiod and light intensity 100 μmol photons m^-1^ s^-1^ for 10 days.

Ten days after germination, Petri dishes were transferred to a temperature**-**controlled incubator, set to −2 ± 0.1°C. To achieve uniform freezing, the Petri dishes were equilibrated at −2°C for 24 h before further lowering the temperature. After equilibration, the temperature of the chamber was progressively lowered at the rate of 1°C per day until the desired sub-zero temperatures were attained. The temperature was monitored by wireless temperature sensor modules (Traceable® Remote sensor module, Fisher Scientific, Ottawa, ON, Canada) placed inside the Petri plates. At each temperature, five Petri plates per treatment were withdrawn from the chamber, thawed at 4°C for 12 h, in the dark, and returned to the original growth conditions (16:8 photoperiod, 22/18°C day/night temperatures). Survival of plants (by degree of chlorosis and shoot damage) was recorded visually, 48 h after returning to the original growth conditions.

#### In vivo freezing assay

The Arabidopsis ecotype Col-0 plants were grown in peat pellets (Jiffy-7®, Jiffy Products Ltd, NB, Canada) in a greenhouse set at 22/18°C day/night temperatures and 16:8 photoperiod with an irradiance of 100 μmol photons m^-1^ s^-1^. Three-week-old plants were used in the experiments. Extract treatments were administered 48 h prior to freezing treatment by irrigating with 1.0 g L^-1^ of ANE or LPC (1.0 g L^-1^ equivalent made up with sterile distilled water) at the rate of 20 ml per plant. The control plants were grown under identical conditions as the plants used in the extract treatment, except they received an equal volume of methanol (100 μl/g equivalent), made up with distilled water, but without the LPC. Forty eight hours after treatment, plants were transferred to a low temperature incubator set to 0°C. Freezing was initiated by spraying ice cold water, and the temperature of the chamber was progressively lowered at the rate of 1°C every 24 h until the desired sub-zero temperature was attained. Temperature was monitored through wireless temperature sensor modules placed at different points inside the freezing cabinet. At each treatment temperature, fifteen randomly selected plants from each treatment were withdrawn from the chamber, thawed at 4°C for 12 h in the dark, and then returned to the original growth conditions of the green house. Two days later, survival of plants was recorded visually according to the degree of chlorosis and leaf damage. Leaf samples, for biochemical assays, collected from plants undergoing the peat pellet freezing tolerance, were flash frozen in liquid nitrogen, ground to fine powder and stored at −80°C until further analysis.

### Proline estimation

To study the role of proline in ANE-mediated freezing tolerance, the proline content was measured in acidic extracts and quantified spectrophotometrically using an acid-ninhydrin reagent with proline as the standard [84]. The proline concentrations of treated plants were compared with untreated controls using Tukey’s HSD test with P≤0.05 and n≥15. Three independent experiments were performed with similar results.

### Proline mutant studies

Petri dish freezing tolerance assays, and peat pellet freezing tolerance assays, were carried out following the methods previously described by Xin and Browse [16], using P5CS mutant plants, to study the role of proline in ANE-mediated freezing tolerance. Arabidopsis proline mutant *p5cs1.1* (SALK_058000) [85] seeds were a generous gift from Dr. Laszlo Szabados (Institute of Plant Biology, Biological Research Center, Szeged, Hungary).

### Transcriptional analysis of proline biosynthesis and degradation genes

The expression of proline biosynthesis genes, *P5CS1 and P5CS2,* and the proline degradation gene, *PRODH* (proline dehydrogenase), was studied by quantitative Real-Time PCR. Leaf samples were taken from −2°C treatments. Leaf tissue from five plants constituted a replicate and there were three replications for each treatment. The leaves were flash frozen in liquid nitrogen, ground with a pre-chilled mortar and pestle and stored at −80°C until further use. Total RNA was isolated using RNA*queous*® Plant RNA isolation Kit (Ambion Inc. Austin, TX) following the manufacturer’s instruction. Ten micrograms of total RNA were treated with DNAase using the TURBO DNA-*free*®kit (Ambion Inc. Austin, TX), and first-strand cDNA was synthesized using the Retroscript® Reverse Transcription Kit (Ambion Inc. Austin, TX). Quantitative Real-time PCR, performed to assess the fold change in *P5CS1 and P5CS2,PRODH* transcript abundance, was carried out in a ‘Step One *Plus*® Real-Time PCR System’ (Applied Biosystems) using ‘Fast Start Universal SYBR Green Master®’ (Roche Diagnostics, Indianapolis, IN) adopting manufacturer’s instructions. Data were analyzed using ‘Step One software V2.0’ with a ‘Relative Standard Curve’ mode. Primer sequences used for this study are provided in the Additional file 2: Table S1.

### Total soluble sugars estimation and sugar mutant studies

Total soluble sugars were estimated by the colorimetric assay developed by Farrar [86]. An Arabidopsis sugar mutant (*sfr4*) (FR67) [87] was used to study the role of soluble sugar accumulation in ANE**-**mediated freezing tolerance. The *sfr4* mutant used in this study was a kind gift from Drs. Matsuo Uemura and Lenn Thorlby (School of Biological Sciences, University of London, UK). The sfr4 mutant is highly sensitive to freezing temperatures as compared to wild-type plants, due to a low sugar content, which leads to a loss of osmotic responsiveness [40]. A peat pellet freezing tolerance assay was carried out to confirm the role of sugar accumulation in ANE-mediated freezing tolerance. Trypan blue stain (3,3-[(3,3-dimethyl-4,4-biphenylylene) bis (azo)] bis(5-amino-4-hydroxy-2,7-naphthalenedisulfonic acid) tetra sodium salt) was used to visualize the extent of freezing-induced tissue damage in extract-treated and control plants. Damaged tissues stained dark blue, while the viable cells did not stain, due to an intact cell membrane barrier [88].

Plant growth conditions, extract treatments and freezing temperatures were as described in the peat pellet freezing assay, as described in Xin and Browse [16]. At each treatment/temperature combination, five plants per treatment were removed from the incubator, thawed at 4°C for 12 h in the dark, and then returned to the normal growth conditions (16 h:8 h day:night cycle; 22/18°C day/night temperatures at an irradiance of 100 μmol photons m^-1^ s^-1^). Two days later, uniform sized leaves, from the second or third whorl, were sampled (by excising at the base of the petiole) and tissue damage was assessed, as described previously by Rate et al. [88]. The area of the damaged tissue was measured using Image-j®image processing and analysis software (Research Services Branch, NIH). For each treatment, at least 15 individual leaves collected from five randomly selected plants (three leaves per plant), were stained and analyzed. Two independent experiments were carried out with similar results.

### Metabolite profiling

#### Global profiling

Global metabolite profiling of freeze-stressed Arabidopsis plants was carried out using a method described by Ward et al. [44], with minor modifications. Total metabolites were extracted from 100 mg of frozen plant tissue using 10 ml methanol (100%), by sonicating for 1 h using an ice/water bath. The extract was decanted into a fresh glass vial and re-extracted with a fresh batch of methanol. The extracts were combined, dried under a stream of nitrogen, and freeze dried to remove residual water. The dried extract was re-suspended in 800 μl deuteriated methanol (Methanol-d_4_) (Cat no 530530, Sigma Aldrich, Oakville, ON), sonicated, and a 750 μl aliquot was transferred into a 5 mm NMR tube (Wilmad-Lab glass, Buena, NJ) for analysis.

NMR spectra were acquired on a Bruker Avance III 600 MHz NMR spectrometer (Bruker Corporation, East Milton, ON), operating at 600.28 MHz ^1^H observation frequency, and a temperature of 25±0.2°C. Spectra were obtained with a gradient broadband inverse 5 mm probe (optimized for ^1^H), auto-tuned, matched and shimmed on each sample, at a flip angle of 30° pulse, followed by a 2.66 s acquisition time and 2 s relaxation delay. Each spectrum consisted of 32 scans of 64 k data points, with a spectral width of 12335.52 Hz. ^1^H NMR chemical shifts in the spectra were referenced to CD_2_H peak of methanol at δ 3.31 ppm, and that peak intensity was also used as a reference for comparison of the level of metabolites level between samples. The signals were acquired, processed and analyzed using TopSpin® NMR data acquisition and processing software (Bruker Biospin Ltd, East Milton, ON) integrated with the spectrometer. The experiment was conducted with three biological replicates and the experiment was repeated twice.

#### Targeted profiling

For targeted metabolite profiling, representative samples used in the NMR experiments above were dried and re-suspended with 20 mM phosphase buffer (pH 7.0), 1 mM NaN3 and 0.49 mM DSS (3-(trimethylsilyl)-1-propanesulfonic acid) in D_2_O. NMR spectra were acquired on the same Bruker Avance III 600 NMR spectrometer, as described above, using a 5 mm inverse gradient probe set to 25.0±0.1°C. 1D NOESY spectra with pre-saturation on water were acquired with 256 scans, an acquisition time of 2.73s, a sweep width of 20 ppm, a mixing time of 100 ms, and relaxation delay of 5s. All spectra were referenced using the internal DSS standard set to 0 ppm. NMR spectra were processed and binned for the region of d 0–10 ppm (water peak region d 4.7-4.83 ppm excluded) within 0.04 ppm chemical shift window by using the NMR software package NMR Suite 5.0 (Chenomx, Edmonton, Canada). The same software package was used to do targeted profiling. Multivariate data analysis (PCA and PLS-DA) was performed by SIMCA-P+ 12.01.0 software package (Umetrics, Umea, Sweden). The experiment had three biological replicates.

### Microarray analysis

#### RNA extraction and cRNA preparation

Total RNA was isolated using an RNA*queous*® Plant RNA isolation kit (Ambion Inc. Austin, TX) following the manufacturer’s instructions. The RNA samples were quantified on a Nanodrop ND1000® (Thermo Scientific, Beverly, MA) and the integrity was checked on an Agilent Bioanalyzer 2100® (Agilent Technologies Canada Inc. Mississauga, ON). CopyRNA was prepared using an Illumina TotalPrep® RNA Amplification kit according to the standard protocol provided by the manufacturer. Briefly, the protocol consisted of first- and second-strand reverse transcription steps, followed by a single, *in vitro* transcription (IVT) amplification step that incorporated biotin-labeled nucleotides. The synthesized cRNA was later purified and quantitated.

#### Hybridization and signal acquisition

Equal quantities of purified cRNA were hybridized to GeneChip® Arabidopsis ATH1 Genome Arrays (Affymetrix Inc. Santa Clara, CA). Subsequent steps included washing, blocking, and streptavadin-Cy3:Cy5 staining. Fluorescence emission was quantitatively detected usingthe iScan® System (Illumina Inc. Hayward, CA) with Illumina Beadstudio® software (Illumina Inc. Hayward, CA). Each treatment had three biological replicates.

### Microarray data analysis

#### Statistical analysis

The raw data were analyzed using Flex Array statistical software (Genome Quebec, Montreal, QC) to obtain a measure of gene expression. Background correction, normalization, and summarization of probe-level gene chip data was performed using the RMA (Robust Multi-chip Average) algorithm in Flex Array. The statistical test used was Wright and Simon’s implementation of the Empirical Bayes method [89]. The gene lists were imported from the annotation file, ATH1-121501.na21.annot.csv (Affymetrix Inc. Santa Clara, CA), available in TIGR database (ATH1-121501).

#### Functional classification of differentially expressed genes

The differentially expressed gene lists were distributed into functional categories using the web based tool, Classification Super-Viewer (http://bar.utoronto.ca/ntools/cgi-bin/ntools_heatmapper_plus.cgi), Bio-Array Resource for Arabidopsis functional genomics, University of Toronto) based on Gene Ontology classes available on the TAIR database (ftp://ftp.arabidopsis.org/home/tair/Ontologies/GeneOntology). Those IDs falling into classification categories, other than unclassified and classification not yet clear-cut, were removed from these two categories. The tool then normalized the number of genes from the experimental data to the number of genes in each class present on the chip, so that differences were more easily perceived. A class score for normalization was calculated based on the following equation [90].

$$\text{Score class}=\frac{{\text{Number in Class}}_{\left(\text{input set}\right)}/{\text{Number in Classified}}_{\left(\text{input set}\right)}}{{\text{Number in Class}}_{\left(\text{reference set}\right)}/{\text{Number Classified}}_{\left(\text{reference set}\right)}}$$

Furthermore, the input set was bootstrapped one hundred times by sampling the input set (with repeats) and then reclassifying each set generated. The standard deviation for the scores generated from the bootstrap sets was displayed along with the normalized class score. In this way classes represented by small numbers of genes on the chip were easily identified [90].

### Pathway analysis

The Pathway Tools Omics Viewer (formerly the Pathway Tools Expression Viewer) (http://gohelle.cirad.fr:1555/expression.html) was used for pathway analysis of genes differentially expressed in the microarray experiment. We followed the standardized *AraCyc*-defined metabolic pathways catalogue [91], which included 1759 *Arabidopsis* enzyme genes, to identify genes in each pathway.

### Heat map and Venn diagram

Heat mapping was perfromed using the web based tool, Heat-mapper *Plus* (http://bar.utoronto.ca/ntools/cgi-bin/ntools_heatmapper_plus.cgi) (Bio-Array Resource for Arabidopsis functional genomics, University of Toronto) and the Venn diagram was generated using the web based tool, Venn Diagram Generator (http://www.pangloss.com/seidel/Protocols/venn.cgi).

### Validation of microarray experiment using RT-PCR

A ‘Two-step RT-PCR’ was used for quantifying transcript yield and comparing differential gene expression. Ten micrograms of total RNA were treated with DNase using the TURBO DNA-*free*®kit (Ambion Inc. Austin, TX), and first-strand cDNA was synthesized using the Retroscript® Reverse Transcription Kit (Ambion Inc. Austin, TX). The cDNA samples were purified with QAIquick® PCR purification Kit (Qiagen Inc. Mississauga, Ontario) and normalized with the *Quantum* RNA® Universal 18S internal standard (Ambion Inc. Austin, TX). A standard PCR reaction mixture was used and the transcripts were amplified using the following profile: 1 cycle of 94°C for 2 min, 30 cycles of 94°C for 30s, 60°C for 30s, 72°C for 1min, followed by 1 cycle of 72°C for 7 min.

## Competing interests

The authors declare no competing interests.

## Authors’ contributions

BP, PR conceived the experiment. BP, PR, JZ, CK, MDH, BB planned the study. PR,JZ, XJ, CK conducted experiments. PR, JZ, CK, MDH, BB, ATC, DH, SK, BP analyzed, interpreted data and wrote the manuscript. All authors read and approved the final manuscript.

## Supplementary Material

Additional file 1 **Figure S1.** Global gene expression of Arabidopsis plants treated with lipophilic fraction of ANE (LPC) during freezing stress and post freezing recovery period. Volcano plot of differential expression pattern during (a) freezing stress and (b) post freezing recovery with ≥1.5 foldchange and P≤0.05 cut off. Scatter plot of log_2_ signal intensities of three replicate samples from LPC**-**treated plants during (c) freezing stress and (d) post freezing recovery. Figure S2. Functional categorization of differentially expressed genes. Normalized frequency (Provart and Zhu, 2003) categories of (a) up, or (c) down-regulated genes in LPC**-**treated plants during freezing. (b&d) functional categorization (b) up or (d) down-regulated genes during freezing according to the actual number of genes. Figure S3. Functional categorization of differentially expressed genes. Normalized frequency (Provart and Zhu, 2003) categories of (a) up, or (c) down-regulated genes in LPC**-**treated plants during post**-**freezing recovery period. (b& d) functional categorization (b) up or (d) down-regulated genes according to the actual number of genes during post**-**freezing recovery period. Figure S4. Heat map of selected differentially expressed genes in LPC treated plants during (FR) freezing stress and (TH) post freezing recovery period. Expression-level change is shown in a color relative to the expression level, as indicated in the color scale bar. Click here for file

Additional file 2 **Table S1.** Primer sets and the PCR conditions used for gene expression studies. This material is available as part of the online article from: http://www.blackwell-synergy.com/doi/. Table S2. A list of common genes which showed the similar pattern of expression (either up-regulated, or down-regulated) during freezing and post**-**freezing thawing period. Table S3. List of genes selected for RT-PCR confirmation of microarray results. Click here for file
