# Supplementary figures and images for: Transcriptional and metabolomic analysis of Ascophyllum nodosum mediated freezing tolerance in Arabidopsis thaliana
Source: BMC Genomics. 2012 Nov 21;13:643. doi: 10.1186/1471-2164-13-643 (PMC3560180; doi:10.1186/1471-2164-13-643)

## Slide 1
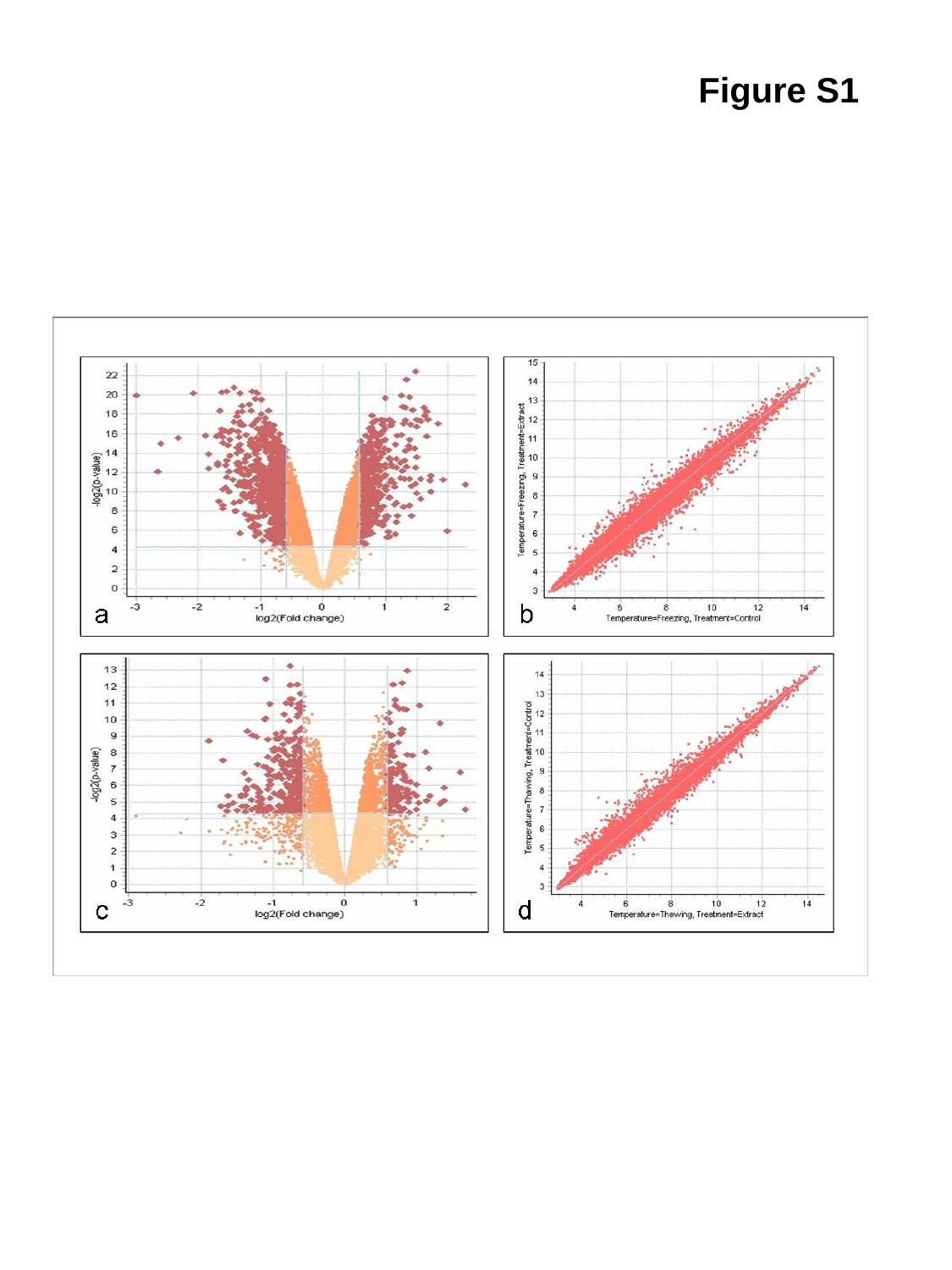

# Figure S1

## Slide 2
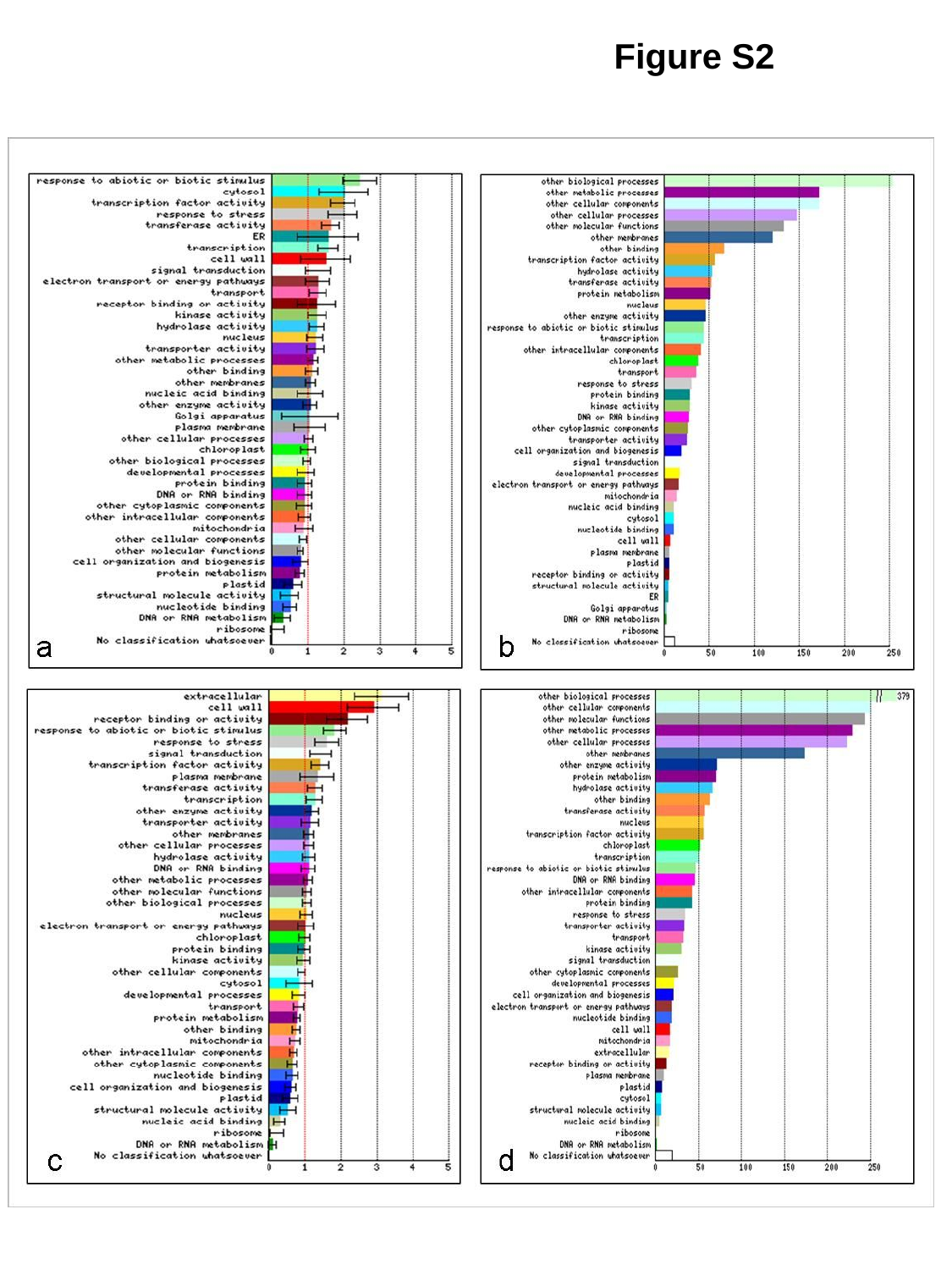

# Figure S2

## Slide 3
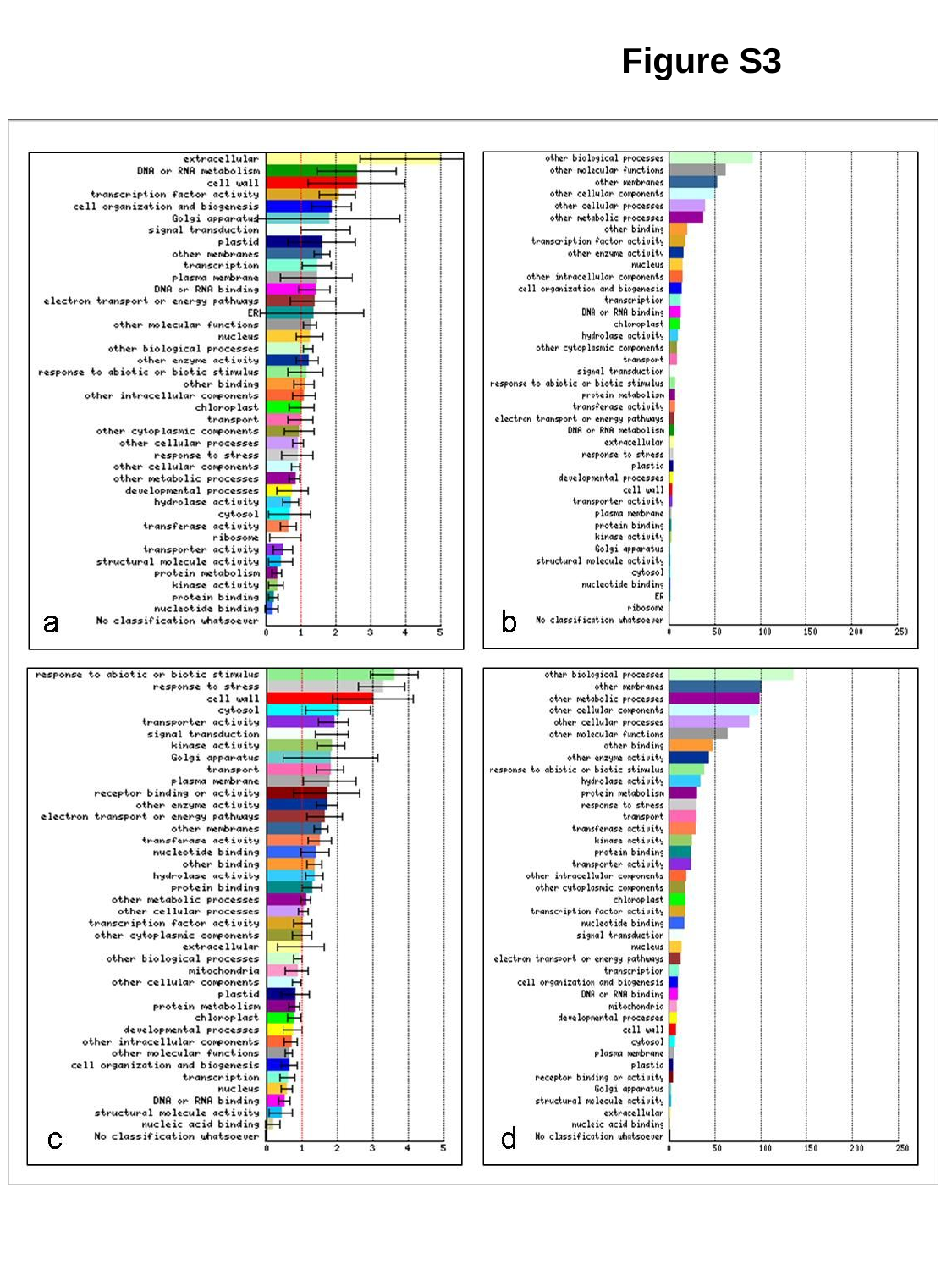

# Figure S3

## Slide 4
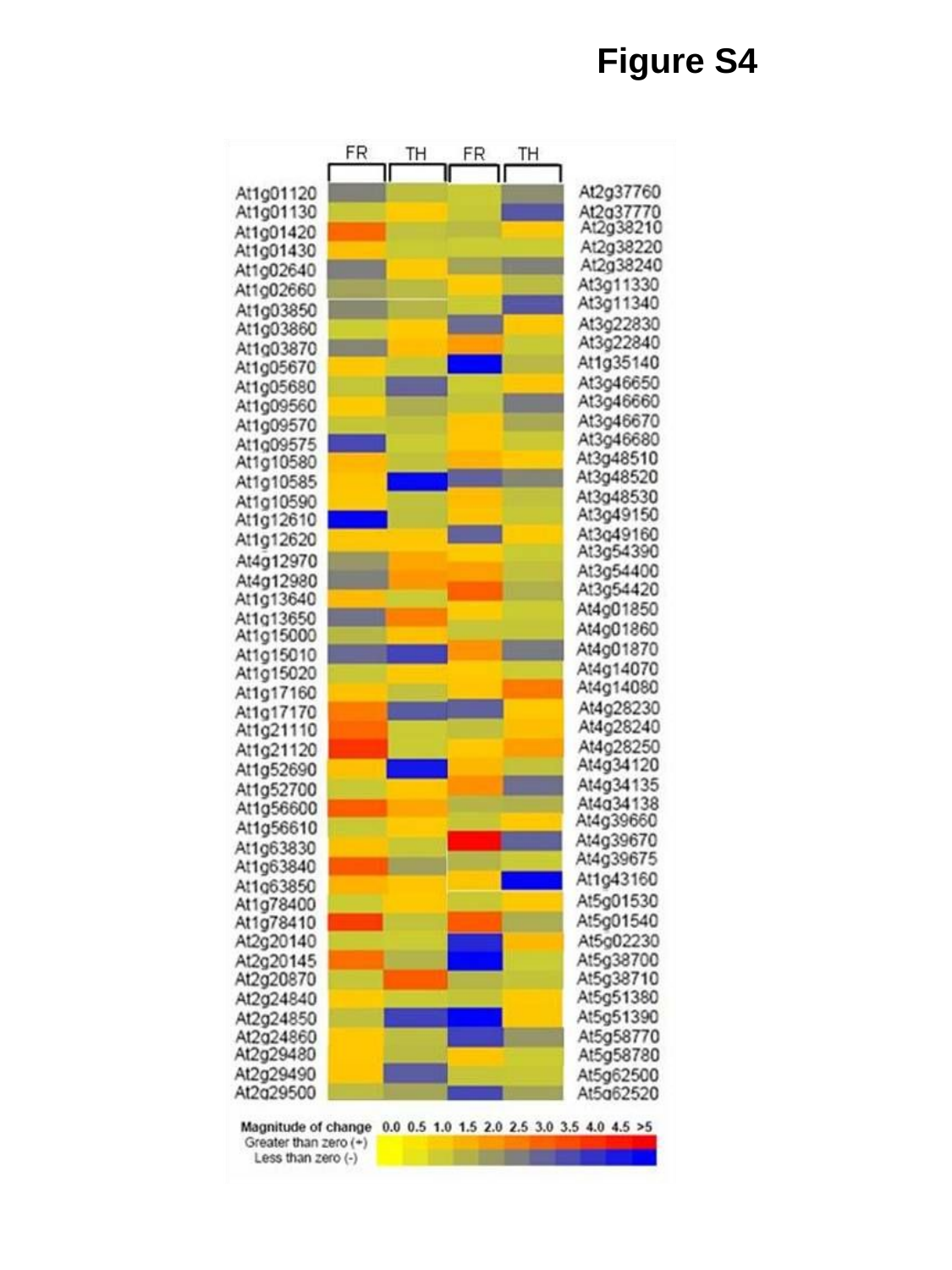

Figure S4

Supplement: Additional file 1 — Figure S1. Global gene expression of Arabidopsis plants treated with lipophilic fraction of ANE (LPC) during freezing stress and post freezing recovery period. Volcano plot of differential expression pattern during (a) freezing stress and (b) post freezing recovery with ≥1.5 foldchange and P≤0.05 cut off. Scatter plot of log2 signal intensities of three replicate samples from LPC-treated plants during (c) freezing stress and (d) post freezing recovery. Figure S2. Functional categorization of differentially expressed genes. Normalized frequency (Provart and Zhu, 2003) categories of (a) up, or (c) down-regulated genes in LPC-treated plants during freezing. (b&d) functional categorization (b) up or (d) down-regulated genes during freezing according to the actual number of genes. Figure S3. Functional categorization of differentially expressed genes. Normalized frequency (Provart and Zhu, 2003) categories of (a) up, or (c) down-regulated genes in LPC-treated plants during post-freezing recovery period. (b& d) functional categorization (b) up or (d) down-regulated genes according to the actual number of genes during post-freezing recovery period. Figure S4. Heat map of selected differentially expressed genes in LPC treated plants during (FR) freezing stress and (TH) post freezing recovery period. Expression-level change is shown in a color relative to the expression level, as indicated in the color scale bar. [file 1471-2164-13-643-S1.ppt]
