# Supplementary material for: Transcriptional and metabolomic analysis of Ascophyllum nodosum mediated freezing tolerance in Arabidopsis thaliana
Source: BMC Genomics. 2012 Nov 21;13:643. doi: 10.1186/1471-2164-13-643 (PMC3560180; doi:10.1186/1471-2164-13-643)
Supplement: Additional file 2 — Table S1. Primer sets and the PCR conditions used for gene expression studies. This material is available as part of the online article from: http://www.blackwell-synergy.com/doi/. Table S2. A list of common genes which showed the similar pattern of expression (either up-regulated, or down-regulated) during freezing and post-freezing thawing period. Table S3. List of genes selected for RT-PCR confirmation of microarray results. [file 1471-2164-13-643-S2.docx]

**Table S1.** Primer sets and the PCR conditions used for gene expression studies.This material is available as part of the online article from: http://www.blackwell-synergy.com/doi/

| **Gene Name** | **Gene Locus** | **Dir** | **Primer Sequence** | **T_m_** |
| --- | --- | --- | --- | --- |
| ***P5CS1*** | AT2G39800 | F | GAGCTAGATCGTTCACGTGCTTT | 57 |
|  |  | R | ACAACTGCTGTCCCAACCTTAAC |  |
| ***P5CS2*** | AT3G55610 | F | GTTAAGCGTATCGTCGTCAAGGTT | 57 |
|  |  | R | CCTAAACGTCCAAGAGCCAATCT |  |
| ***ProDH*** | AT3G30775 | F | TCACAACCACTGAGCTAAAGTGAGA | 57 |
|  |  | R | CGATGACGCTGTATCTTGTGATG |  |

**Table S2.** A list of common genes which showed the similar pattern of expression (either up-regulated or down-regulated) during freezing and post freezing thawing period

| **No** | **Locus** | **FC**  FR | **Gene Ontology Class** | **Gene Annotation Detail** |
| --- | --- | --- | --- | --- |
|  |  | TH |  |  |
| **Common Up-Regulated Genes** | | | | |
| 1 | At1g48750 | 1.5 | Lipid Binding,  Endomembrane system | protease inhibitor/seed storage/lipid transfer protein (LTP) family protein |
|  |  | 1.7 |  |  |
| 2 | At2g47880 | 1.7 | endomembrane system | glutaredoxin family protein; thiol-disulfide exchange intermediate activity |
|  |  | 1.7 |  |  |
| **Common Down-Regulated Genes** | | | | |
| 1 | At1g17420 | 2.4 | response to wounding,  jasmonic acid biosynthetic process | LOX3__LOX3 (Lipoxygenase 3); iron ion binding / lipoxygenase/ metal ion binding / oxidoreductase, acting on single donors with incorporation of molecular oxygen, incorporation of two atoms of oxygen |
|  |  | 1.6 |  |  |
| 2 | At1g59500 | 2.3 | response to auxin stimulus | GH3.4__GH3.4; indole-3-acetic acid amidosynthetase |
|  |  | 1.6 |  |  |
| 3 | At1g01560 | 1.9 | response to abscisic acid stimulus;  signal transduction | ATMPK11__ATMPK11 (Arabidopsis thaliana MAP kinase 11) |
|  |  | 1.5 |  |  |
| 4 | At1g09500 | 1.7 | lignin biosynthetic process | cinnamyl-alcohol dehydrogenase family / CAD family |
|  |  | 3.1 |  |  |
| 5 | At1g66160 | 2.2 | protein ubiquitination | U-box domain-containing protein |
|  |  | 1.6 |  |  |
| 6 | At1g61340 | 2.9 | Molecular function unknown | F-box family protein |
|  |  | 1.9 |  |  |
| 7 | At1g71330 | 1.7 | ATPase activity, coupled to transmembrane movement of substances | ATNAP5__ATNAP5 (Arabidopsis thaliana non-intrinsic ABC protein 5) |
|  |  | 1.8 |  |  |
| 8 | At1g79270 | 1.6 | Biological process unknown | ECT8__ECT8 (evolutionarily conserved C-terminal region 8) |
|  |  | 1.5 |  |  |
| 9 | At3g51860 | 2.0 | membrane of vacuole with cell cycle-independent morphology | CAX3_ATHCX1_CAX1-LIKE__CAX3 (cation exchanger 3); cation:cationantiporter |
|  |  | 2.0 |  |  |
| 10 | At2g39030 | 1.5 | N-acetyltransferase activity; metabolic process | GCN5-related N-acetyltransferase (GNAT) family protein |
|  |  | 3.2 |  |  |
| 11 | At3g23920 | 1.5 | cellulose biosynthetic process | BMY7_TR-BAMY__BAM1/BMY7/TR-BAMY (BETA-AMYLASE 1); beta-amylase |
|  |  | 1.5 |  |  |
| 12 | At4g13900 | 1.5 | signal transduction | pseudogene, similar to NL0D, similar to Cf-4A protein (Lycopersiconesculentum) |
|  |  | 1.5 |  |  |
| 13 | At4g37530 | 1.7 | response to oxidative stress; endomembrane system | peroxidase, putative |
|  |  | 1.9 |  |  |
| 14 | At4g31800 | 1.8 | regulation of transcription, DNA-dependent;  defense response to fungus | WRKY18__WRKY18 (WRKY DNA-binding protein 18); transcription factor |
|  |  | 2.0 |  |  |
| 15 | At4g28140 | 1.8 | regulation of transcription, DNA-dependent | AP2 domain-containing transcription factor, putative |
|  |  | 2.0 |  |  |
| 16 | At4g18010 | 1.7 | inositol-polyphosphate 5-phosphatase activity; | IP5PII__IP5PII (INOSITOL POLYPHOSPHATE 5-PHOSPHATASE II); inositol-polyphosphate 5-phosphatase |
|  |  | 2.5 |  |  |
| 17 | At5g13320 | 1.6 | detection of fungus;  molecular function unknown | PBS3_GDG1__PBS3 (AVRPPHB SUSCEPTIBLE 3) |
|  |  | 1.8 |  |  |
| 18 | At5g58770 | 3.7 | dolichol biosynthetic process | dehydrodolichyldiphosphate synthase, putative / DEDOL-PP synthase, putative |
|  |  | 2.0 |  |  |
| 19 | At5g13220 | 2.5 | Molecular function unknown | JAS1_JAZ10_TIFY9__JAS1/JAZ10/TIFY9 (JASMONATE-ZIM-DOMAIN PROTEIN 10) |
|  |  | 1.8 |  |  |
| 20 | At5g19240 | 1.6 | Molecular function unknown | Identical to Uncharacterized GPI-anchored protein At5g19240 precursor [Arabidopsis Thaliana] (GB:Q84VZ5;GB:Q8H7A4); similar to unknown protein [Arabidopsis thaliana] (TAIR:AT5G19230.1); similar to unknown [Populustrichocarpa] (GB:ABK94712.1) |
|  |  | 1.6 |  |  |

**Table S3.** List of genes selected for RT-PCR confirmation of microarray results

| **No** | **Gene Locus** | **Dir** | **Primer Sequence** | **T_m_** | **# Cyl** |
| --- | --- | --- | --- | --- | --- |
| 1 | At4g39670 | F | GAGCCATTCGCGTAACCTAA | 60 | 30 |
|  |  | R | GTTCTAACGGCCCACGTATG |  |  |
| 2 | At1g78410 | F | AGCCGATGAAGGTTGTGTTC | 60 | 30 |
|  |  | R | CGATTTTACTACCGCCATCG |  |  |
| 3 | At1g63840 | F | TTCCGGTGGGATATTCTGAG | 60 | 30 |
|  |  | R | TGAGTCAGGTTTGGTCAACG |  |  |
| 4 | At1g56600 | F | GCTGGTGCTAAACCTTGGAG | 60 | 30 |
|  |  | R | TGTTGCTTCTTGTGGCTGTC |  |  |
| 5 | At1g73330 | F | AGTCGACGGAGGTGCTTACA | 60 | 30 |
|  |  | R | GAGTTGCTGCCTTCTGGAAC |  |  |
| 6 | At2g20870 | F | TTCCCAAGTCCCCTAAGACC | 60 | 30 |
|  |  | R | GGATTCCCCCACCAGTAAGT |  |  |
| 7 | At2g40610 | F | GGAGCTTGTGGCTATGGAAA | 60 | 30 |
|  |  | R | CTGTGACGGTGATGGTTGAC |  |  |
| 8 | At1g74670 | F | ATGTGGAGGACAATGCACAA | 60 | 30 |
|  |  | R | GGACATTTTGGTCCACCTTG |  |  |
| 9 | At4g22490 | F | CCACCATTTCCCTCATCATC | 60 | 30 |
|  |  | R | GCCAGCTGTAACATTGGCTA |  |  |
| 10 | At5g20740 | F | GCTTGTGGACTGTGTGGAGA | 60 | 30 |
|  |  | R | ACACGTGTCGTCATCCGTAA |  |  |
